# Clinical applications of reticulocyte hemoglobin indices, including reticulocyte hemoglobin equivalent (Ret-He) and reticulocyte hemoglobin content (CHr), among pregnant and postpartum women: A scoping review and evidence map

**DOI:** 10.1371/journal.pone.0362481

**Published:** 2026-11-13

**Authors:** Miki Ohtsuki, Takuma Ohsuga, Kohei Ida, Kaori Tsuyuki, Miho Egawa, Mayumi Toyama, Yoshimitsu Takahashi, Takeo Nakayama, Masaki Mandai

**Affiliations:** 1 Department of Gynecology and Obstetrics, Kyoto University Graduate School of Medicine, Kyoto, Japan; 2 Department of Health Informatics, Kyoto University School of Public Health, Kyoto, Japan; 3 Department of Implementation Science in Public Health, Kyoto University School of Public Health, Kyoto, Japan; USUHS: Uniformed Services University of the Health Sciences, UNITED STATES OF AMERICA

## Abstract

Early detection of iron deficiency during pregnancy and the postpartum period, a common condition associated with adverse maternal and neonatal outcomes, remains challenging with conventional iron markers. Reticulocyte hemoglobin indices, including reticulocyte hemoglobin equivalent and reticulocyte hemoglobin content, may enable earlier detection of iron-restricted erythropoiesis. We conducted a scoping review to map the clinical and research applications of these indices in pregnant and postpartum women. Following Joanna Briggs Institute methodology and PRISMA-ScR guidance, we searched PubMed, CENTRAL, Web of Science, and Ichushi Web for studies published from January 1, 1990, to May 31, 2025, and performed an updated search on April 21, 2026, for newly added or indexed records. Eligible studies involved pregnant women or women within 3 months postpartum in whom reticulocyte hemoglobin indices were evaluated for clinical or research purposes. We also constructed an evidence map to visualize the distribution of evidence and research gaps. Thirty-five studies were included. Evidence was most developed for diagnostic accuracy for iron deficiency and associations with iron-related biomarkers. Reticulocyte hemoglobin indices generally showed good diagnostic performance, although reported cut-off values varied widely across studies. Data from mid-to-late pregnancy remained limited. Studies using reticulocyte hemoglobin indices as indicators of response to iron therapy are increasing. Some studies examined longitudinal changes and reference values across pregnancy and the postpartum period; however, these studies were methodologically limited. Three studies examined associations between maternal reticulocyte hemoglobin indices and neonatal parameters, whereas no study evaluated maternal clinical outcomes. Overall, reticulocyte hemoglobin indices appear promising for assessing iron status and monitoring response to iron therapy in perinatal populations. However, substantial heterogeneity in study design, reference standards, and analytical platforms, together with limited longitudinal and outcome-oriented evidence, currently constrains broader clinical implementation.

## Introduction

Iron deficiency remains the most common single-nutrient deficiency worldwide, affecting approximately 29% of non-pregnant women and 38% of pregnant women [1]. During pregnancy, iron requirements increase substantially owing to fetal growth, expansion of maternal plasma volume, and placental metabolic activity [2]. When iron stores are inadequate, depletion occurs rapidly, leading to iron deficiency (ID) even before anemia develops, with iron deficiency anemia (IDA) representing the final hematological manifestation of this process. Maternal ID and IDA during the perinatal period have been associated with adverse outcomes, including low birth weight, impaired long-term neurocognitive development in children, and maternal postpartum depression [3–5].

However, routine antenatal care in most countries relies primarily on complete blood counts, which detect anemia but fail to identify the earlier, pre-anemic stages of ID. Although serum ferritin can assess iron stores, its interpretation during pregnancy is complicated by inflammation and hemodilution, and standardized diagnostic thresholds are lacking [6]. Conventional red blood cell indices are widely available, but respond slowly to changes in iron status because of the approximately 120-day lifespan of erythrocytes.

Reticulocyte hemoglobin equivalent (Ret-He) and reticulocyte hemoglobin content (CHr), which assess the hemoglobin content of reticulocytes, have emerged as potential biomarkers that may help address these limitations. CHr was initially developed by Technicon (later Bayer and Siemens) and approved by the U.S. Food and Drug Administration in 1997, followed by approval of Ret-He, developed by Sysmex, in 2005 [7]. Other parameters—including MCHr (mean cellular hemoglobin content of reticulocytes; Abbott), RHCc (reticulocyte hemoglobin cellular content; Horiba), and RHE (reticulocyte hemoglobin expression; Mindray) —provide similar assessments. Although no standardized collective term exists, this review collectively refers to the reticulocyte hemoglobin indices as Ret-He/CHr.

Hemoglobin synthesis occurs primarily during early erythropoiesis, and insufficient iron supply at this stage leads to reduced hemoglobin content in reticulocytes. As reticulocytes mature into erythrocytes within a few days, Ret-He/CHr reflects functional iron availability for erythropoiesis over the preceding 3–4 days, enabling early detection of iron-restricted erythropoiesis [8,9]. These indices may decrease in inflammatory states when inflammatory cytokines and hepcidin restrict iron absorption and macrophage iron release, thereby limiting iron availability for erythropoiesis. However, unlike ferritin, they are not acute-phase reactants and are not expected to increase directly in response to inflammation. They may also be less directly affected by hemodilution than concentration-based iron markers. Ret-He/CHr can be measured rapidly and at low cost, and these indices have demonstrated clinical utility in diagnosing IDA, monitoring treatment response, and managing ID across diverse clinical settings [10–15].

Given the marked fluctuations in iron status during pregnancy and the postpartum period, Ret-He/CHr may offer particular clinical value in these populations, although its use in routine obstetric practice remains limited. Therefore, this scoping review aimed to map and synthesize the current evidence on the use of Ret-He/CHr during pregnancy and the postpartum period, with particular attention to their diagnostic applications, use in monitoring response to iron therapy, and potential clinical relevance in obstetric care.

## Methods

### Study design and protocol registration

We conducted this scoping review to map the scope and characteristics of the clinical and research applications of Ret-He/CHr in pregnant and postpartum women. The review followed the Joanna Briggs Institute (JBI) methodology and was reported in accordance with the Preferred Reporting Items for Systematic Reviews and Meta-Analyses Extension for Scoping Reviews. The protocol was prospectively registered in the Open Science Framework (OSF; https://osf.io/2fkp8/; Registration https://doi.org/10.17605/OSF.IO/2FKP8) [16]. The interval between the original search and final manuscript preparation was prolonged; hence, an updated search was conducted on April 21, 2026, and an amendment note, along with the updated search strategies, was uploaded to the associated OSF project on April 30, 2026. A further amendment documenting the supplementary validation search for additional analyzer-specific terminology was uploaded to the OSF project on June 27, 2026.

### Search strategy and selection criteria

We searched PubMed, the Cochrane Central Register of Controlled Trials (CENTRAL), Web of Science, and Ichushi Web (Japan Medical Abstracts Society database). The search strategy combined terminology for reticulocyte hemoglobin indices with terms related to pregnancy and postpartum contexts and was developed with input from an academic medical librarian to account for variation in terminology across analyzers (e.g., Ret-He, CHr, and RHe) and perinatal care settings. The full search strategies are provided in S1 Appendix. Since CHr measurement technology was developed in the 1990s, the original search included records published from January 1, 1990, to May 31, 2025. The updated search used the same databases, search concepts, and eligibility criteria, and was restricted to records newly added or indexed after the original search period. A supplementary validation search using additional analyzer-specific terms, including RHCc and RHE, was also conducted on June 13, 2026, which identified no additional eligible studies. Gray literature was restricted to conference abstracts or similar records identified through database searches, provided sufficient methodological information could be retrieved to allow data extraction. Eligibility criteria were defined using the Population–Concept–Context (PCC) framework: pregnant women or women within 3 months postpartum (Population), purpose and clinical utility of Ret-He/CHr measurement (Concept), and no geographic restrictions within the specified publication period (Context). The eligible sources included original research articles, case reports, and conference abstracts or other non-peer-reviewed publications published in English or Japanese. Review articles, animal studies, studies limited to analytical or technical aspects without clinical evaluations, publications in other languages, and reports without accessible full texts were excluded.

The search results were imported into Rayyan for deduplication. Two reviewers independently screened titles and abstracts, and full texts were assessed when eligibility was unclear. Discrepancies were resolved through discussion with a third reviewer. The study selection process is presented in the Preferred Reporting Items for Systematic Reviews and Meta-Analyses Extension for Scoping Reviews flow diagram.

### Data analysis

The following data were extracted: bibliographic information (authors, publication year, and journal); country of study; study design and analytical methods; hematology analyzer used; participant characteristics (sample size and gestational age or postpartum timing); primary outcomes; and key findings related to Ret-He/CHr and iron metabolism-related parameters. Data extraction was performed independently by two reviewers using a standardized form, and agreement was evaluated during the pilot extraction phase. Disagreements were resolved through discussion with a third reviewer.

The extracted data were synthesized descriptively and mapped according to the timing of Ret-He/CHr measurement, reference standards for iron deficiency or related conditions, applied Ret-He/CHr diagnostic cut-off values, and analytical methods. To visualize temporal and geographic trends in the literature, the included studies were aggregated by country and publication period and presented graphically. As this was a scoping review, no formal risk-of-bias assessment was conducted; however, clinical and methodological experts were consulted, as needed, during interpretation.

### Evidence map

To visualize the distribution of existing evidence and research gaps, we constructed an evidence map using a four-dimensional bubble plot [17,18]. Data from each included study were categorized accordingly. Bubbles were labeled with the first author, and publication years were added when multiple studies by the same author were included. An initial version of the evidence map was prepared by one reviewer and refined through discussions with the review team.

The four dimensions were defined as follows: The X-axis (pregnancy stage) represented the timing of data collection: first trimester (≤13 weeks’ gestation), second trimester (14–27 weeks), third trimester (≥28 weeks), and postpartum. Studies contributing data from multiple pregnancy stages were represented by separate bubbles for each stage. Longitudinal data from the same cohort were indicated by grouping the corresponding bubbles within a rectangular outline. Studies that did not distinguish between trimesters were categorized as “antenatal (unspecified)” studies.

The Y-axis (study purpose) reflected how Ret-He/CHr was used or evaluated in each study based on the reported study objectives and outcome measures. Study purposes were classified into seven categories: diagnostic accuracy for ID or anemia; associations with iron-related parameters (including ferritin, other iron markers, and blood cell indices); associations with inflammatory markers; associations with perinatal outcomes; reference ranges; longitudinal changes; and response to iron therapy. When a study addressed multiple purposes, each analysis was presented as a separate bubble.

Bubble size was proportional to the number of women included in each analysis. Bubble color represented the study design: prospective observational studies (blue), retrospective observational studies (purple), and interventional studies (yellow).

To align the evidence map with the aim of this review, studies focusing on hemoglobinopathies or using Ret-He/CHr–based indices (e.g., the Thomas plot) as reference standards rather than index tests were included in the review but not visualized in the evidence map. Protocol-only publications without reported outcome data were also included but not visualized.

## Results

A total of 789 records were identified in the original search and 31 in the updated search. After removal of 120 and 6 duplicates, respectively, 669 records from the original search and 25 from the updated search were screened. The study selection process is shown in Fig 1. In total, 35 studies were included in the review: 31 from the original search and 4 from the updated search.

**Fig 1:**
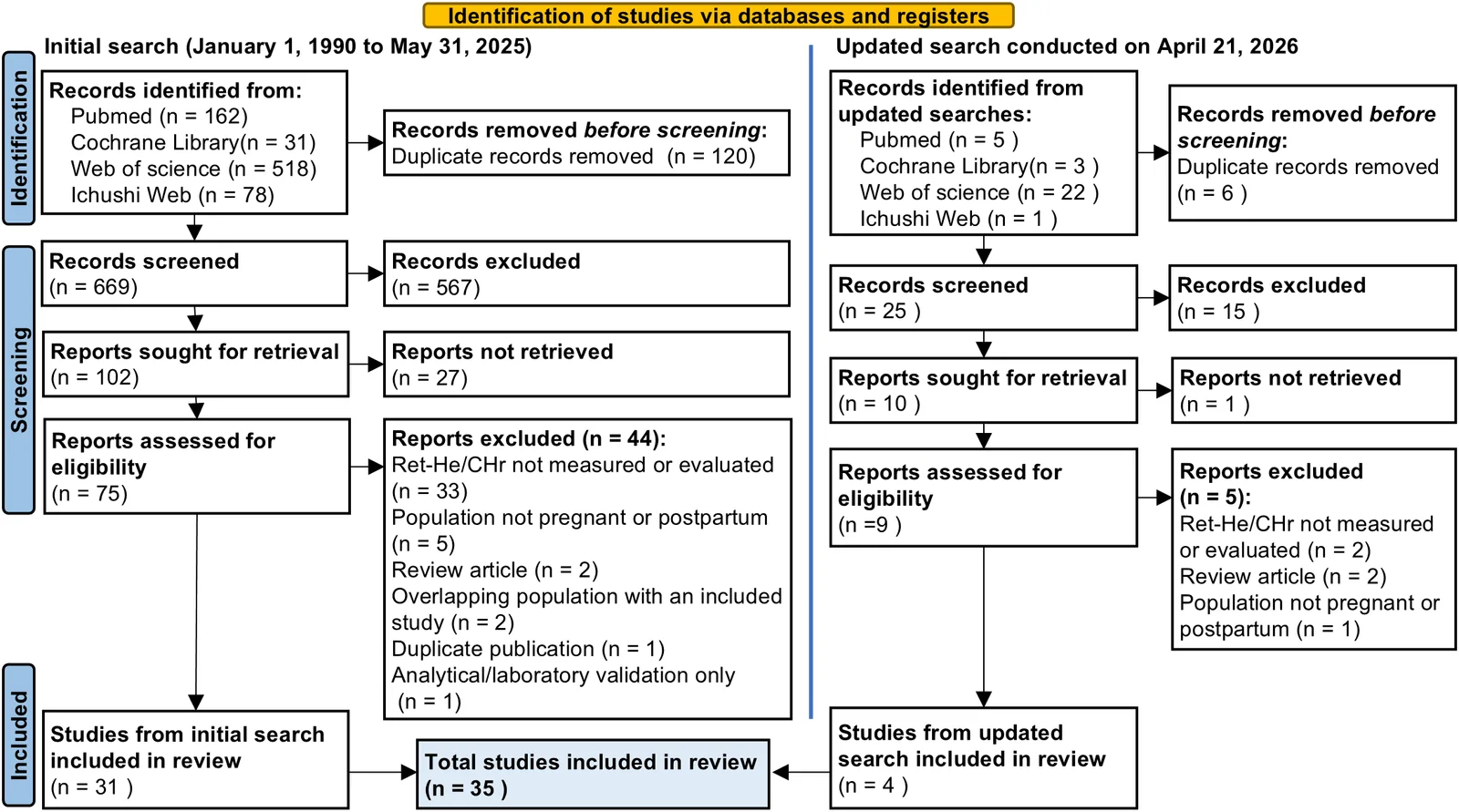
PRISMA flow diagram. Flow diagram showing the identification, screening, eligibility assessment, and inclusion of studies from both the original and updated searches in this scoping review.

Reasons for exclusion at the full-text stage are provided in S2 Table. Table 1 presents an overview of the included studies [19–53]. Sample size refers to the number of women contributing to Ret-He/CHr analyses rather than the total number enrolled.

**Table 1:** Overview of the included studies.

First author (year)	Country	Study design	N	Parameter (analyzer/ manufacturer)
Breymann (1999) [19]	Switzerland	Intervention	4	MCHr (Technicon H3/ Bayer Diagnostics)
Richter (1999) [20]	Switzerland	Prospective longitudinal	82	CHr (Technicon H3 RTC/··)
Krafft (2003) [21]	Switzerland	Prospective cross-sectional	64	CHr (Advia/ Bayer AG)
Krafft (2005) [22]	Switzerland	Intervention	52	MCHr (Technikon H3/ Bayer Diagnostics)
Ervasti (2007) [23]	Finland	Prospective cross-sectional	202	CHr (Advia 120/ Bayer Diagnostics)
Ervasti (2008) [24]	Finland	Prospective cross-sectional	210	CHr (Advia 120/ Bayer Diagnostics)
Bjørke-Monsen (2012) [25]	Norway	Prospective longitudinal	104	CHr (Advia 120/ Bayer Diagnostics)
Ramakers (2012) [26]	Netherlands	Prospective longitudinal	195	CHr (Advia 120/ Siemens Healthcare Diagnostics)
Schoorl (2012) [27]	Netherlands	Prospective longitudinal	25	Ret-He (Sysmex XE2100/ Sysmex Corp.)
Urrechaga (2014) [28]	Spain	Prospective cross-sectional	70	RetHe (Sysmex XE 500/ Sysmex Corp.)
Holm (2015) [29]	Denmark	Intervention (protocol)	200	CHr (··)
Weyers (2016) [30]	South Africa	Prospective cross-sectional	103	RET-He (SysmexXR-2100/ Sysmex Corp.)
Amstad Bencaiova (2017) [31]	Switzerland	Intervention	50	CHr (Advia/ Bayer Diagnostics)
Levy (2018) [32]	South Africa	Prospective cross-sectional	102	Ret-He (Sysmex XN 9000/ Sysmex Corp.)
Demmers (2019) [33]	Netherlands	Prospective cross-sectional	926	MCHr (CELL-DYN Sapphire/ Abbott Diagnostics)
Yu (2019) [34]	China	prospective longitudinal	133	Ret-He (Sysmex XE-2100/ Sysmex Corp.)
Kumar (2020) [35]	India	Prospective cross-sectional	155	Ret-He (Sysmex-XN 1000/ Sysmex Corp.)
Markova (2020) [36]	Denmark	Intervention (protocol)	200	CHr (··)
Priddee (2020) [37]	United Kingdom	Prospective cross-sectional	1966	RET-He (··)
Babacheva (2022) [38]	Greece	Prospective cross-sectional	200	MCHr (Alinity Hq/ abbott)
Kumar (2022) [39]	India	Prospective cross-sectional	155	Ret-He (Sysmex-XN 1000/ Sysmex Corp.)
Santana (2022) [40]	United States	Retrospective	37	RET-He (··)
Schoorl (2022) [41]	Netherlands	Prospective longitudinal	39	Ret-He (Sysmex XN 9000/ Sysmex Corp.)
Bó (2023) [42]	Brazil	Prospective cross-sectional	60	RET-He (Sysmex XN10 Roche/ Sysmex Corp.)
Griffin (2023) [43]	United States	Prospective longitudinal	499	RetHb (··)
Singh (2023) [44]	India	Prospective cross-sectional	166	Ret-Hb (Sysmex XN 1000/ Sysmex Corp.)
Casper (2024) [45]	United States	Prospective cross-sectional	165	RET-He (··)
Sahithya (2024) [46]	India	Prospective cross-sectional	280	Ret-He (Sysmex XN 1000/ Sysmex Corp.)
Shu (2024) [47]	China	Prospective cross-sectional	724	RHE (Mindray BC-6900/ Mindray Corp.)
Afifah (2025) [48]	Indonesia	Intervention	40	RET-He (··/ Sysmex Corp.)
Haizler-Cohen (2025) [49]	United States	Prospective cross-sectional	196	Ret-He (··)
Casper (2025) [50]	United States	Prospective cross-sectional	65	RET-He (··)
Akshaykirthan (2025) [51]	India	Prospective longitudinal	315	Ret-Hb (··/ Sysmex Corp.)
Patki (2025) [52]	India	Intervention	108	RET-He (··)
Pekar (2025) [53]	United States	Prospective longitudinal	177	RET-He (Sysmex XN/ Sysmex Corp.)

Key characteristics of the included studies: first author, years, country of study, study design, sample size (N), and reticulocyte hemoglobin-based parameters with the corresponding analyzers and manufacturers used to assess Ret-He/CHr. Publication years were based on journal issue dates, except for Pekar et al. [53], for which the online publication year (2025) was used because the article was available before the updated literature search. Abbreviations: Ret-He, reticulocyte hemoglobin equivalent; CHr, reticulocyte hemoglobin content.

Of the included studies, 28 were prospective observational studies (including 9 longitudinal cohort studies), 1 was a retrospective observational study, and 7 were interventional studies, 2 of which were protocol studies.

Fig 2 presents the temporal and geographical distribution of the included studies. Early research was concentrated in Europe, with broader geographic representation emerging over time. Most studies were conducted in Europe (n = 17), followed by Asia (n = 9), North America (n = 6), Africa (n = 2), and South America (n = 1).

**Fig 2:**
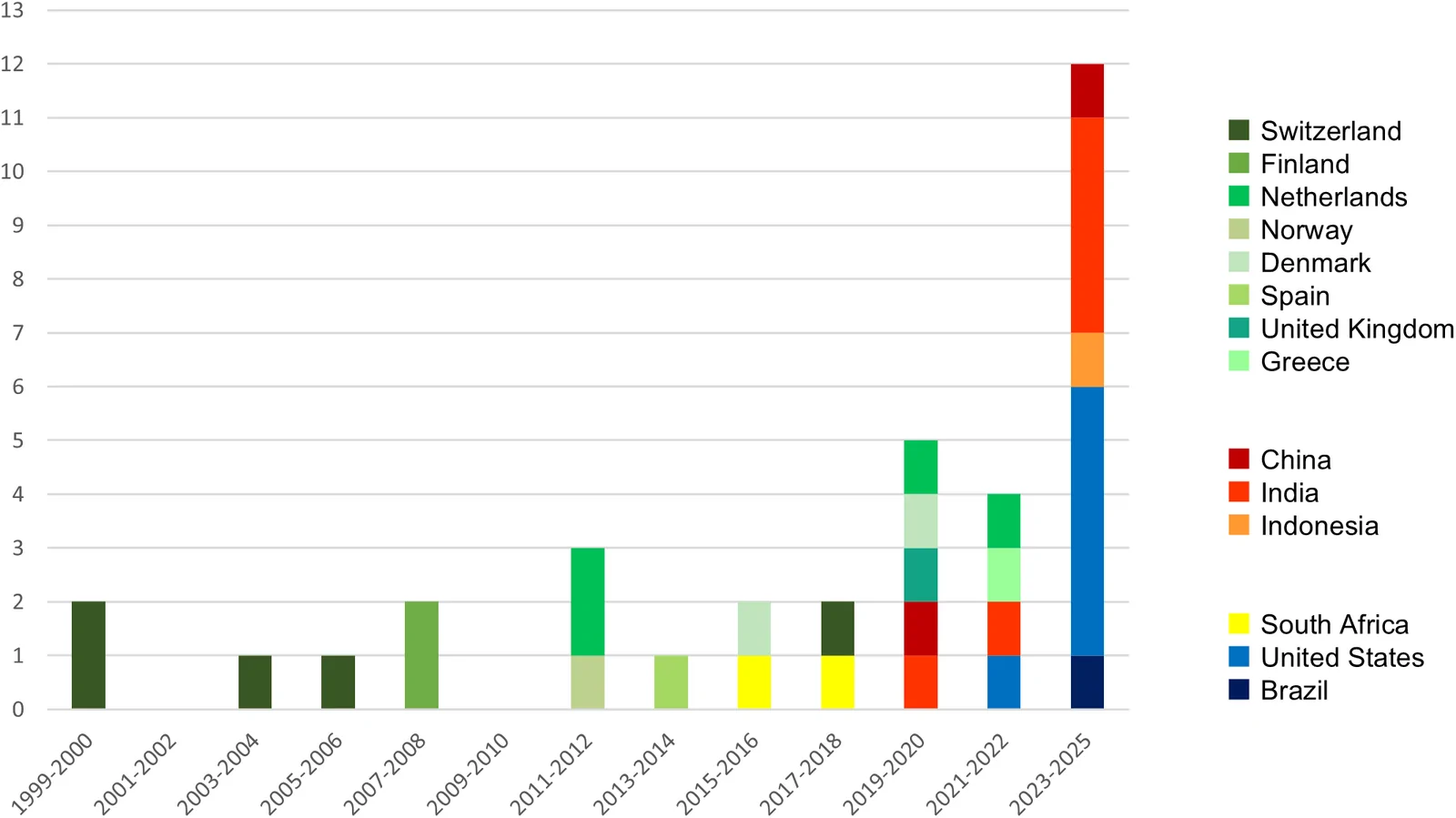
Geographical distribution of included studies by year. Colors indicate geographical regions: green shades represent European countries, red shades represent Asian countries, and other colors represent studies conducted in other regions.

The distribution of evidence across pregnancy stage, study purpose, sample size, and study design is presented in the evidence map (Fig 3). The evidence map, which included 30 of the 35 eligible studies with available outcome data suitable for visualization, showed that diagnostic accuracy for iron deficiency and associations with iron-related biomarkers were the most frequently investigated topics, whereas evidence from mid-to-late pregnancy was limited. Among the included studies, Ret-He/CHr was also used as an indicator of response to iron therapy, and several studies described changes in Ret-He/CHr values across pregnancy and the postpartum period, including longitudinal analyses within the same cohort. However, many of these studies had notable methodological limitations, which are described in detail in the Discussion. Three studies evaluated the associations between maternal Ret-He/CHr and neonatal parameters; no study assessed associations with maternal clinical outcomes.

**Fig 3:**
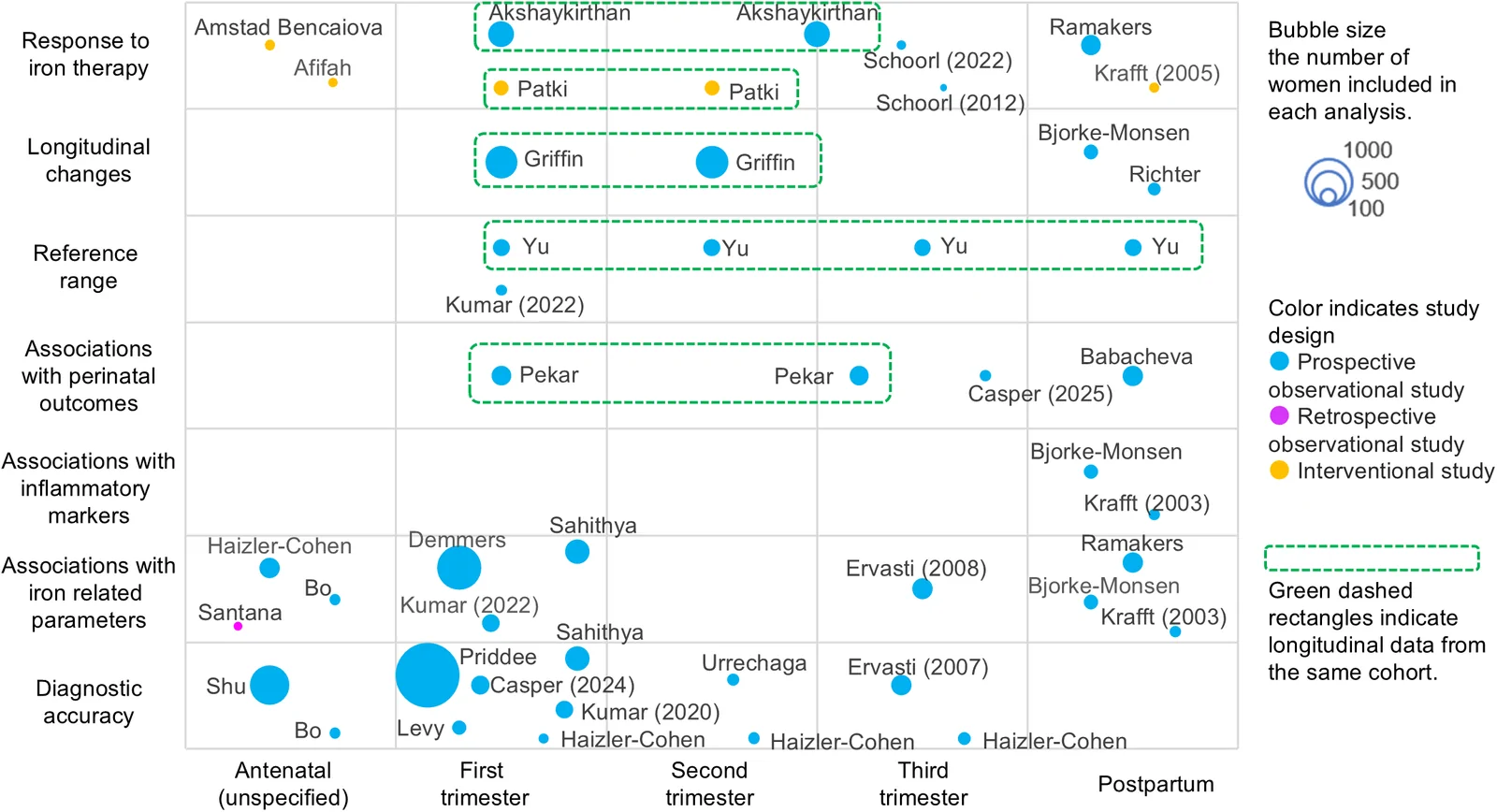
Evidence map. Each bubble represents an analytical unit derived from an included study and is labeled by the first author (and publication year when necessary). The x-axis indicates pregnancy stage. “Antenatal (unspecified)” refers to studies in which the timing of Ret-He/CHr measurement was reported as during pregnancy, but not stratified by trimester. When a single study reported data from multiple stages, each stage is shown as a separate bubble, and longitudinal data from the same cohort are enclosed within a rectangular outline. The y-axis denotes the analytical purpose of Ret-He/CHr use or evaluation. The bubble size is proportional to the sample size, and the bubble color indicates the study design.

The following sections summarize the findings in line with the analytical objectives for Ret-He/CHr. Terminology for Ret-He/CHr varies across analyzers and studies; therefore, study-specific parameter names are reported as used in the original articles where relevant, whereas Ret-He/CHr is used as a collective term when findings are summarized across studies. Detailed methodological and statistical information for each analytical objective is provided in the Supporting Information Tables.

### Diagnostic accuracy studies

Eleven studies evaluated the diagnostic accuracy of Ret-He/CHr, nine of which focused on its performance for detecting anemia and/or iron deficiency (Table 2). Diagnostic targets, reference standards, and reported cut-off values varied across studies. Additional details, including analyzer platforms and subgroup-specific analyses, are provided in S3–S6 Tables.

**Table 2:** Diagnostic performance of Ret-He/CHr for anemia and iron deficiency.

Author (year)	Country	N	Timing	Diagnostic target	Cut-off	AUC	Comparator
Ervasti (2007) [23]	Finland	202	admission for delivery	IDA†	30.5	0.95	sTfR: 0.79
Haizler-Cohen (2025) [49]	United States	196	antenatal (unspecified)	IDA‡	··	0.82	··
Priddee (2020) [37]	United Kingdom	1966	first trimester	Ferritin < 30 μg/L	··	0.72	Hb: 0.70
Bó (2023) [42]	Brazil	60	antenatal (unspecified)	Ferritin < 13 μg/L	··	0.73	Hb: 0.59
Sahithya (2024) [46]	India	280	first trimester	Ferritin < 20 μg/L	32.25	0.99	··
Shu (2024) [47]	China	724	antenatal (unspecified)	Ferritin < 15 μg/L	29.85	0.84	MCV: 0.654
Haizler-Cohen (2025) [49]	United States	196	antenatal (unspecified)	Ferritin < 30 μg/L	34.0	0.65	··
Haizler-Cohen (2025) [49]	United States	196	antenatal (unspecified)	Ferritin < 15 μg/L	··	0.72	··
Ervasti (2007) [23]	Finland	202	admission for delivery	TSAT ≤ 11%	31.9	0.79	ferritin: 0.77
Urrechaga (2014) [28]	Spain	70	20 weeks’ gestation	TSAT < 15%	29.9	0.79	MCH: 0.71
Casper (2024) [45]	United States	165	≤ 14 weeks’ gestation	TSAT < 20%	33.2	0.77	ferritin: 0.64
Levy (2018) [32]	South Africa	102	first trimester	ID (iron markers)	31.2	0.81	MCH: 0.78
Kumar (2020) [35]	India	155	first trimester	ID (blood cell indices)	27.8	0.93	ferritin: 0.76

Summary of diagnostic targets, cut-off values for Ret-He/CHr (pg), AUC, and comparator parameters reported in studies evaluating Ret-He/CHr. Antenatal (unspecified) refers to measurements reported during pregnancy without trimester stratification.

Haizler-Cohen et al. [49] report the diagnostic performance using two different ferritin cut-offs; the results are shown as separate analyses. Comparator refers to the parameter with the highest reported AUC in the same study.

IDA†, Hb ≤12.2 g/dL, MCV ≤86 fL, and ferritin ≤11 μg/L

IDA‡, Hb < 11 g/dL (second trimester: < 10.5 g/dL) and ferritin <30 μg/L.

ID (iron markers), serum iron <9 μmol/L, TSAT <20%, or ferritin <30 μg/L

ID (Blood cell indices), MCV < 83 fL, and MCH < 27 pg.

Abbreviations: Ret-He, reticulocyte hemoglobin equivalent; CHr, reticulocyte hemoglobin content; AUC, area under the receiver operating characteristic curve; sTfR, soluble transferrin receptor; IDA, iron deficiency anemia; ID, iron deficiency; Hb, hemoglobin; MCV, mean corpuscular volume; TSAT, transferrin saturation; MCH, mean corpuscular hemoglobin.

Two studies assessed the diagnostic performance of Ret-He/CHr for IDA, and both demonstrated high accuracy (AUC > 0.8).

For ID, reference standards included serum ferritin, transferrin saturation (TSAT), and red blood cell indices. Five studies used ferritin-based definitions of ID, with Ret-He/CHr generally performing well at lower ferritin thresholds (e.g., < 20 μg/L) [42,46,47]. In contrast, accuracy was more variable at higher cut-offs. For example, Haizler-Cohen et al. [49] reported reduced accuracy for mild ID defined as ferritin < 30 μg/L (AUC 0.65), whereas Priddee et al. [37] reported an AUC of 0.72 at the same threshold. Because Haizler-Cohen et al. also conducted trimester-stratified analyses, this study was represented by multiple bubbles in the evidence map.

Studies using TSAT as the reference standard reported diagnostic performance comparable to ferritin and superior to red blood cell indices. In a TSAT-based analysis stratified by body mass index, Ret-He outperformed ferritin among individuals with obesity [45]. When composite reference standards that combined iron markers or red blood cell indices were used, Ret-He/CHr consistently showed good diagnostic performance (AUC > 0.8).

One study evaluated Ret-Hb for β-thalassemia rather than iron deficiency or anemia, and therefore described separately; this study reported an AUC of 0.823 (95% CI, 0.65–0.99) [44].

### Associations between Ret-He/CHr and other parameters

Thirteen studies evaluated the association between Ret-He/CHr and other parameters. Detailed correlation coefficients and statistical measures are provided in S7–S9 Tables. Among six studies examining ferritin using correlation analyses, most reported moderate associations, whereas Bjørke-Monsen et al. [25] identified a nonlinear dose-response relationship between CHr and ferritin using generalized additive models, with a marked change in slope around a ferritin level of approximately 20 µg/L. Demmers et al. [33] also reported that lower MCHr values were significantly associated with low ferritin concentrations in logistic regression analyses. Associations with other iron-related markers or erythrocyte indices varied in strength across studies, but the direction of association was generally consistent with established iron deficiency physiology. In contrast, no significant associations were reported between Ret-He/CHr and inflammatory markers (S8 Table). Three studies evaluated the associations between maternal Ret-He/CHr and neonatal parameters (S9a and S9b Tables). In their studies examining maternal–neonatal correlations using Spearman’s correlation, Babacheva et al. [38] and Casper et al. [50] reported weak-to-moderate positive associations. In contrast, Pekar et al. [53] evaluated the predictive value of maternal RET-He for neonatal anemia and low neonatal RET-He using receiver operating characteristic curve analysis. Maternal RET-He did not predict neonatal anemia but showed good accuracy for predicting low neonatal RET-He (AUC > 0.8). Babacheva et al. and Pekar et al. also reported no significant differences in maternal Ret-He/CHr according to maternal diabetes status.

### Reference ranges and longitudinal changes

Five studies reported Ret-He/CHr values during pregnancy and/or the postpartum period, including two reference range studies and three studies evaluating longitudinal changes (S10 and S11 Tables).

The reference values were derived under different methodological conditions. Kumar et al. [39] restricted their analyses to first-trimester women without anemia, ID, or iron supplementation, reporting a median Ret-He value of 29 pg, whereas Yu et al. [34] reported percentile-based values across pregnancy and the postpartum period without exclusion or stratification by iron status or iron supplementation.

The remaining studies evaluated longitudinal changes in Ret-He/CHr. Most studies did not account for iron supplementation or iron deficiency status; however, Griffin et al. [43] stratified analyses according to iron deficiency status and reported mean RetHb values of approximately 34 pg in women without iron deficiency in the first and second trimesters.

### Response to iron therapy

Ret-He/CHr was used as an indicator of response to iron therapy in both observational and interventional studies, with changes assessed following iron-based therapy (Table 3). Additional details regarding analyzer platforms, treatment or intervention characteristics, and pre- and post-treatment Ret-He/CHr values are provided in S12 and S13a–e Tables. One interventional study in patients with thalassemia was included but is summarized only in the Supplementary Tables owing to its distinct clinical context [19].

**Table 3:** Response to iron therapy assessed using Ret-He/CHr.

Author (year)	Country	N	Timing	Subgroup/ Comparison	Δ	Sig.
Ramakers (2012) [26] †	Netherlands	195	postpartum	baseline CHr < 28 pg	1.9	↑
Ramakers (2012) [26] †	Netherlands	195	postpartum	baseline CHr ≥ 28 pg	−1	↓
Schoorl (2012) [27] †	Netherlands	25	third trimester	IDE (baseline Ret-He < 29.6 pg)	4.7	↑
Schoorl (2022) [41] †	Netherlands	39	27–30 weeks’ gestation	IDE (baseline Ret-He < 29.8 pg)	6	↑
Akshaykirthan (2025) [51]†	India	315	12–16 weeks’ gestation	Single cohort (all participants had IDA)	4.5	↑
Krafft (2005) [22] ‡	Switzerland	52	postpartum	intervention (iron supplement)	0.9	NR
Krafft (2005) [22] ‡	Switzerland	52	postpartum	control (placebo)	0.3	NR
Amstad Bencaiova (2017) [31] ‡	Switzerland	50	19–38 weeks’ gestation	IDA (baseline CHr < 28 pg)	6.8	↑
Amstad Bencaiova (2017) [31] ‡	Switzerland	50	19–38 weeks’ gestation	ACD + ID (baseline CHr > 28 pg)	3.2	↔
Afifah (2025) [48] ‡	Indonesia	40	14–32 weeks’ gestation	intervention (baseline RET-He < 32.1 pg)	2.3	↔
Afifah (2025) [48] ‡	Indonesia	40	14–32 weeks’ gestation	intervention (baseline RET-He ≥ 32.1 pg)	−1.1	↓
Afifah (2025) [48] ‡	Indonesia	40	14–32 weeks’ gestation	control (baseline RET-He < 32.1 pg)	−1.2	↔
Afifah (2025) [48] ‡	Indonesia	40	14–32 weeks’ gestation	control (baseline RET-He ≥ 32.1 pg)	−1.2	↓
Patki (2025) [52] ‡	India	108	13–16 weeks’ gestation	intervention (SunActive™ Fe)	5.32	↑
Patki (2025) [52] ‡	India	108	13–16 weeks’ gestation	active comparator (ferrous ascorbate)	4.25	↑

This table summarizes observational and interventional studies that evaluated changes in the Ret-He/CHr in response to iron therapy and/or concomitant interventions during pregnancy or the postpartum period. Changes are presented according to treatment allocation and/or baseline Ret-He/CHr stratification.

In Afifah et al. [48], all women received iron therapy. The intervention group additionally received a fiber supplement, whereas the control group received milk powder. N indicates the total number of women included in each study (subgroup-specific sample sizes are provided in S11 and S12 Tables). Δ indicates the mean or median change (after minus before) in Ret-He or CHr, as reported. Sig. indicates statistical significance: ↑ statistically significant increase; ↓ statistically significant decrease; ↔ no statistically significant change; NR, not reported.

† observational study; ‡ interventional study.

Abbreviations: Ret-He, reticulocyte hemoglobin equivalent; CHr, reticulocyte hemoglobin content; IDE, iron-deficient erythropoiesis; IDA, iron deficiency anemia; ACD, anemia of chronic disorders.

Four observational studies evaluated the responses to iron therapy during pregnancy or the postpartum period [26,27,41,51]. In these studies, Ret-He/CHr increased after oral iron supplementation among women with low baseline values, whereas among women with higher baseline CHr values, small but statistically significant decreases were observed in one study [26].

Among the four interventional studies with available outcome data, Ret-He/CHr was used to evaluate responses to iron-based interventions with or without concomitant treatments, such as erythropoietin, in pregnant or postpartum women. In two studies, Ret-He/CHr increased following intervention in women with iron-deficiency anemia [31,52]. In contrast, no increase was observed among women with anemia of chronic disease who had higher baseline CHr values [31]. The remaining studies reported no significant overall changes [22], except for small but statistically significant decreases in subgroups with higher baseline Ret-He values in one study [48].

## Discussion

To the best of our knowledge, this scoping review provides the first comprehensive mapping of the use of Ret-He/CHr in pregnant and postpartum women. The findings suggest that Ret-He/CHr may be a useful biomarker for assessing iron status during pregnancy and the postpartum period. The evidence map further highlights the limited available data from mid-to-late pregnancy.

Although diagnostic accuracy was the most frequently examined application, the included studies also addressed reference ranges, longitudinal changes during pregnancy and the postpartum period, responses to iron therapy, and associations with maternal or perinatal outcomes. Therefore, we chose a scoping review approach because the field is characterized by substantial heterogeneity in biological context (measurement timing across the gestational and postpartum periods), methodology (reference standards, ferritin thresholds, and outcome definitions), and technical factors (analyzer platforms and terminology). Accordingly, the primary aim of this study was to map the scope and structure of the evidence rather than to estimate the pooled diagnostic performance, which explains why no formal risk-of-bias assessment or meta-analysis was undertaken.

Evidence evaluating the diagnostic performance of Ret-He/CHr for ID and IDA is expanding, with many studies reporting a performance comparable or superior to that of conventional iron indices. However, reported cut-off values vary widely, ranging from approximately 27.8 to 34.0 pg. This variability likely reflects differences in population characteristics, analytical platforms, and reference standards.

Ethnic or ancestry-related differences in Ret-He/CHr have not been well established. However, conditions that impair erythropoiesis or hemoglobin synthesis, including nutritional deficiencies such as vitamin B12 or folate deficiency and hemoglobinopathy carrier states such as thalassemia, may affect Ret-He/CHr values independently of iron deficiency [19,44,54]. Therefore, the interpretation of Ret-He/CHr should take into account the characteristics of the target population, including nutritional status and the prevalence of hemoglobinopathy carrier states or other conditions affecting erythropoiesis.

Moreover, although agreement across hematology analyzers has generally been reported [55,56], a UK NEQAS Haematology pilot study identified systematic differences between manufacturers, with reported inter-platform differences of up to approximately 6 pg [57]. Even within the same manufacturer, improvements in analytical performance over time may affect measured values, as illustrated by differences between earlier systems, such as H*3, and newer ADVIA analyzers [9,58]. These factors underscore the importance of considering analyzer-specific calibration when interpreting Ret-He/CHr values. Notably, internationally standardized cut-off values for Ret-He/CHr have not been established, even in nonpregnant populations. The cut-off values identified in this review align broadly with existing guidelines, including recommendations from UK-based organizations such as the National Institute for Health and Care Excellence and the British Society for Haematology (29 pg or 25–30 pg) [11,14], as well as prior reports placing cut-offs in the high-20s to low-30s pg range [10,59–61].

Interpretation of Ret-He/CHr during pregnancy is further challenged by the lack of consensus regarding thresholds for established iron markers, particularly ferritin levels. A World Health Organization guideline [62] subsequently highlighted, with reference to a 2017 systematic review [6], that commonly used ferritin cut-offs in pregnancy (such as < 12 µg/L or < 15 µg/L) are often adopted without clear justification. In this context, two studies included in this review suggest that Ret-He/CHr may provide complementary information for reassessing ferritin thresholds in pregnant and postpartum women. Bjǿrke-Monsen et al. [25] reported a nonlinear dose-response relationship between CHr and ferritin, indicating a possible threshold for iron-deficient erythropoiesis. Weyers et al. [30] applied the Thomas plot incorporating Ret-He to evaluate a ferritin cut-off of 30 µg/L. Although limited, these findings highlight a potential role for Ret-He/CHr in refining operational definitions of iron deficiency.

Associations between Ret-He/CHr and other laboratory parameters have also been explored. Correlations with ferritin levels were generally moderate, consistent with the fact that Ret-He/CHr reflects iron availability for erythropoiesis rather than iron stores, which may be influenced by inflammation. Notably, previous studies have shown that correlations between ferritin and Ret-He are typically strongest within iron-deficient ranges [59]. Similar patterns have been observed in several included studies. Associations with mean corpuscular volume were generally moderate to strong, although differences in the temporal window captured by these indices may contribute to variability across studies. Only three studies evaluated the associations between Ret-He/CHr and inflammatory markers, and none reported clear associations. This may reflect the fact that Ret-He/CHr is not an acute-phase reactant like ferritin, although inflammation may still indirectly lower these indices through hepcidin-mediated iron restriction. Three studies evaluated the associations between maternal Ret-He/CHr and neonatal parameters, while no study evaluated the associations with maternal clinical outcomes.

Iron metabolism changes dynamically throughout pregnancy and the postpartum period, highlighting the importance of trimester-specific and postpartum reference values. Although several studies have reported longitudinal changes or reference ranges across gestation, none have evaluated the associations between Ret-He/CHr trajectories and clinically relevant outcomes, such as subsequent anemia. Furthermore, many studies did not stratify analyses by iron supplementation status, despite evidence that Ret-He/CHr responds rapidly to iron therapy [63]. Diagnostic accuracy studies are frequently limited to early pregnancy or fail to distinguish between trimesters, resulting in a paucity of data in mid-to-late gestation.

Beyond screening for ID, Ret-He/CHr may also be useful for monitoring response to iron therapy. While expert recommendations commonly assess treatment response at approximately 4 weeks in pregnant women with IDA [64], Ret-He/CHr has been shown to respond earlier than conventional markers [63]. In this review, several studies reported increases in Ret-He/CHr following iron therapy, particularly among individuals with low baseline values. However, most assessments occurred at 4 weeks, leaving shorter-term dynamics insufficiently characterized. Studies reporting non-significant or attenuated responses should be interpreted with caution, as factors such as impaired iron absorption or dietary influences may have contributed to the lack of measurable change [22,48].

In addition to identifying evidence gaps, this review provides insights into the temporal and geographic trends in the literature. Early studies were largely conducted in Europe, where CHr was initially developed, with broader geographic representation emerging as reticulocyte hemoglobin measurements became available on analyzers from multiple manufacturers. However, the coexistence of multiple terms for these reticulocyte hemoglobin indices may have contributed to conceptual ambiguity and to challenges in cross-study comparisons. Greater standardization of parameter nomenclature and measurement reporting may facilitate international comparisons and support broader clinical application of Ret-He/CHr.

In clinical practice, Ret-He/CHr may be feasible to implement in settings where hematology analyzers capable of measuring reticulocyte hemoglobin indices are already available, as these values can be obtained rapidly and at a relatively low cost as part of an extended complete blood count or reticulocyte analysis. Potential care pathways include early pregnancy screening, monitoring after the initiation of iron therapy, and postpartum reassessment. Ret-He/CHr may be particularly useful in obstetric clinics, primary care settings, or resource-limited areas where immediate access to biochemical iron markers, such as ferritin, is difficult. In tertiary or hospital-based obstetric care, these indices may also serve as adjunctive markers when ferritin is difficult to interpret because of hemodilution, inflammation, or other clinical conditions. However, Ret-He/CHr should currently be regarded as adjunctive rather than replacement markers for ferritin and hemoglobin, because their optimal role in clinical decision-making pathways remains insufficiently defined.

A key strength of this review is its systematic mapping of the clinical and research applications of Ret-He/CHr, which identified both areas of accumulating evidence and important gaps. Evidence was most developed for the detection of iron deficiency, while several included studies evaluated Ret-He/CHr for monitoring the response to iron therapy. However, current evidence remains insufficient to support universal implementation of Ret-He/CHr in routine perinatal care. The reported cut-off values varied substantially across studies, probably reflecting differences in target populations, gestational timing, reference standards, and analyzer platforms. In addition, longitudinal evidence linking Ret-He/CHr values or trajectories to clinically relevant maternal or neonatal outcomes remains limited. Therefore, before Ret-He/CHr can be incorporated into routine perinatal care, further studies are needed to establish population-, pregnancy-stage-, and analyzer-specific thresholds; evaluate whether Ret-He/CHr improves the prediction of clinically relevant outcomes; and determine how these indices should be integrated with existing markers such as hemoglobin and ferritin. Pragmatic longitudinal studies embedded in antenatal and postpartum care pathways are particularly important for clarifying whether Ret-He/CHr-guided assessment can improve perinatal outcomes.

This scoping review had some limitations. First, as a scoping review, we did not formally assess the methodological quality or risk of bias of the included studies. Therefore, the strength of the evidence for specific clinical applications of Ret-He/CHr could not be formally evaluated. Second, substantial heterogeneity in study design, populations, reference standards, and outcome definitions limited direct comparability across studies. Third, publication and reporting biases may have influenced the available evidence. Studies with favorable diagnostic performance, significant associations, or positive treatment responses may be more likely to be published or reported in detail. Consequently, the evidence map may overrepresent applications with positive findings. Finally, data charting, categorization of study purposes, and construction of the evidence map involved a degree of interpretive judgment, although these processes were conducted by multiple reviewers to enhance rigor.

## Conclusions

Ret-He/CHr may be a useful adjunctive tool for identifying iron deficiency and monitoring the response to iron therapy in pregnant and postpartum women. However, substantial evidence gaps remain, particularly regarding population-, pregnancy-stage-, and analyzer-specific thresholds, longitudinal changes, and clinically relevant maternal and neonatal outcomes. Further well-designed longitudinal and pragmatic studies that integrate detailed clinical data are needed to clarify the role of reticulocyte hemoglobin indices in routine perinatal care.

## Supporting information

S1 Appendix Full search strategies. The full search strategies for the original, updated, and supplementary validation searches are presented. The updated searches used database-specific record creation or indexing date limits to identify records newly added after the original search period. Parallel publication-date-limited searches were performed as a validation check and identified no additional unique records. A supplementary validation search was also conducted using additional analyzer-specific terms related to reticulocyte hemoglobin indices.
(DOCX)

S2 Table Reports excluded after full-text assessment and reasons for exclusion. Listed are all reports excluded after full-text review, including reports not retrieved and those excluded following eligibility assessment. When multiple records were identified as referring to the same study, they were consolidated and listed once.
(DOCX)

S3 Table Diagnostic performance of Ret-He/CHr for iron deficiency anemia or microcytic anemia. The diagnostic accuracy studies evaluating Ret-He/CHr for iron deficiency anemia (IDA) or microcytic anemia are summarized. Information on study population (N), timing of blood sampling (T), diagnostic definitions, cut-off values, and measures of diagnostic performance is provided to complement the main results presented in Table 2. Sensitivity and specificity are reported as provided in the original studies. Comparator indices refer to alternative hematologic or iron-related markers evaluated within the same study. Antenatal (unspecified) refers to measurements reported during pregnancy without trimester stratification. Abbreviations: AUC, area under the receiver operating characteristic curve; SE, standard error; CI, confidence interval; CHr, cellular hemoglobin in reticulocytes; RET-He/Ret-He, reticulocyte hemoglobin equivalent; Hb, hemoglobin; MCV, mean corpuscular volume; sTfR, soluble transferrin receptor. (Abbreviation expansions are presented as described in the original studies.).
(DOCX)

S4 Table Diagnostic performance of Ret-He/CHr for iron deficiency (ID) defined by serum ferritin. The diagnostic accuracy studies evaluating Ret-He/CHr for ID defined by serum ferritin are summarized. Information on study population (N), timing of blood sampling (T), ferritin thresholds used to define ID, cut-off values for Ret-He/CHr, and measures of diagnostic performance is provided to complement the main results presented in Table 2. Subgroup analyses reflect secondary or stratified analyses reported within individual studies and were not uniformly defined across all included studies. Sensitivity and specificity are reported as provided in the original studies. Comparator indices refer to alternative hematologic or iron-related markers evaluated within the same study. Antenatal (unspecified) refers to measurements reported during pregnancy without trimester stratification. Abbreviations: AUC, area under the receiver operating characteristic curve; CI, confidence interval; RET-He, RHE, and Ret-He, reticulocyte hemoglobin equivalent; Ret-He†, hemoglobin content of reticulocytes; Hb, hemoglobin; MCHC, mean corpuscular hemoglobin concentration; MCV, mean corpuscular volume; MCH, mean corpuscular hemoglobin. (Abbreviation expansions are presented as described in the original studies. Superscripts were added where the same abbreviation was used with different term expansions.).
(DOCX)

S5 Table Diagnostic performance of Ret-He/CHr for iron deficiency (ID) defined by transferrin saturation (TSAT). The diagnostic accuracy studies evaluating Ret-He/CHr for ID defined by TSAT are summarized. Information on study population (N), timing of blood sampling (T), TSAT thresholds used to define ID, cut-off values for Ret-He/CHr, and measures of diagnostic performance is provided to complement the main results presented in Table 2. Subgroup analyses reflect secondary or stratified analyses reported within individual studies and were not uniformly defined across all included studies. Sensitivity and specificity are reported as provided in the original studies. Comparator indices refer to alternative hematologic or iron-related markers evaluated within the same study. Abbreviations: AUC, area under the receiver operating characteristic curve; CI, confidence interval; CHr, cellular hemoglobin in reticulocytes; RetHe, reticulocyte hemoglobin equivalent; RET-He, reticulocyte hemoglobin concentration; Hb, hemoglobin; MCV, mean corpuscular volume; MCH, mean corpuscular hemoglobin; sTfR, soluble transferrin receptor. (Abbreviation expansions are presented as described in the original studies.).
(DOCX)

S6 Table Diagnostic performance of Ret-He/CHr for iron deficiency (ID) defined using composite reference standards. The diagnostic accuracy studies evaluating Ret-He/CHr for ID using composite reference standards are summarized. Information on study population (N), timing of blood sampling (T), composite definitions of ID, cut-off values for Ret-He/CHr, and measures of diagnostic performance is provided to complement the main results presented in Table 2. Sensitivity and specificity are reported as provided in the original studies. Comparator indices refer to alternative hematologic or iron-related markers evaluated within the same study. Abbreviations: AUC, area under the receiver operating characteristic curve; CI, confidence interval; Ret-He, reticulocyte hemoglobin content; Hb, hemoglobin; MCV, mean corpuscular volume; MCH, mean corpuscular hemoglobin; TSAT, transferrin saturation. (Abbreviation expansions are presented as described in the original studies.).
(DOCX)

S7 Table Associations between Ret-He/CHr and ferritin. The reported associations between Ret-He/CHr and ferritin are summarized. Associations were evaluated using heterogeneous statistical approaches across studies, including correlation analyses (Spearman’s rs), generalized additive models (GAM), and regression-based analyses. Reported results are presented as described by the original authors and reflect study-specific definitions and analytical methods. Antenatal (unspecified) refers to measurements reported during pregnancy without trimester stratification. N, number of women included in each study; T, timing of blood sampling. Abbreviations: MCHr, reticulocyte hemoglobin content; CHr, reticulocyte hemoglobin content; Ret-He, reticulocyte hemoglobin equivalent; RET-He, reticulocyte hemoglobin. (Abbreviation expansions are presented as described in the original studies.).
(DOCX)

S8 Table Associations between Ret-He/CHr and other iron-related markers, erythrocyte indices, or inflammatory markers. Other iron-related markers include sTfR, TSAT, EPO, and erythrocyte indices (e.g., Hb, MCV, HRC, IRF). Inflammatory markers include CRP and neopterin. Reported statistical measures include correlation coefficients (reported as r values, with the specific correlation method not always specified) and coefficients of determination (R²), as described in the original studies. Results are presented as reported by the original authors and reflect study-specific definitions and analytical methods. Antenatal (unspecified) refers to measurements reported during pregnancy without trimester stratification. N, number of women included in each study; T, timing of blood sampling. Abbreviations: CHr, reticulocyte hemoglobin content; CHr†, cellular hemoglobin in reticulocytes; RET-He, reticulocyte hemoglobin; RET-He†, reticulocyte hemoglobin equivalent; Ret-He, hemoglobin content of reticulocytes; sTfR, soluble transferrin receptor; TSAT, transferrin saturation; EPO, erythropoietin; Hb, hemoglobin; HRC, hypochromic red blood cell percentage; MCV, mean corpuscular volume; IRF, immature reticulocyte fraction; CRP, C-reactive protein. (Abbreviation expansions are presented as described in the original studies. Superscripts were added where the same abbreviation was used with different term expansions.).
(DOCX)

S9 Table Associations between maternal Ret-He/CHr and neonatal parameters. Studies evaluating associations between maternal Ret-He/CHr and neonatal iron-related parameters are summarized. In Babacheva et al., neonatal measurements were obtained from a peripheral arterial blood sample collected within 12 h after birth, whereas Casper et al. and Pekar et al. used umbilical cord blood samples. **A. Correlations between maternal and neonatal Ret-He/CHr.** Studies that evaluated maternal–neonatal correlations using Spearman’s correlation analysis are summarized. N, number of women included in each study; T, timing of blood sampling. Abbreviations: MCHr, mean reticulocyte hemoglobin content; RET-He, reticulocyte hemoglobin content. (Abbreviation expansions are presented as described in the original studies.). **B. Predictive performance of maternal RET-He for neonatal anemia and low neonatal RET-He [53].** The predictive value of maternal RET-He for neonatal anemia and low neonatal RET-He, assessed using receiver operating characteristic curve analysis, is summarized. N, number of women included in each study; T, timing of blood sampling. Abbreviations: RET-He, reticulocyte hemoglobin equivalent; HgB, hemoglobin; AUC, area under the receiver operating characteristic curve; CI, confidence interval. (Abbreviation expansions are presented as described in the original studies.).
(DOCX)

S10 Table Reference range studies of Ret-He/CHr during pregnancy and the postpartum period. Studies that report reference ranges for Ret-He/CHr during pregnancy and the postpartum period are summarized. Reported values are heterogeneous across studies and are not intended to represent unified reference intervals. Statistical measures are reported as presented in the original studies. Stratification or exclusion indicates whether analyses accounted for iron deficiency (ID) status and/or iron supplementation. N, number of women included in each study. 95%RV, 95% reference interval (2.5th–97.5th percentiles). Abbreviations: Ret-He, reticulocyte hemoglobin; Ret-He†, reticulocyte hemoglobin equivalent; Hb, hemoglobin; MCV, mean corpuscular volume. (Abbreviation expansions are presented as described in the original studies. Superscripts were added where the same abbreviation was used with different term expansions.).
(DOCX)

S11 Table Longitudinal changes in Ret-He/CHr during pregnancy and postpartum. Studies reporting longitudinal changes in Ret-He/CHr during pregnancy or postpartum are summarized. Statistical measures are reported as presented in the original studies. Stratification or exclusion indicates whether analyses accounted for iron deficiency (ID) status and/or iron supplementation; for example, Griffin et al. stratified analyses by ID status using a ferritin threshold of < 30 µg/L. N, number of women included in each study; wk, week; mo, month. Abbreviations: IQR, interquartile range; CHr, reticulocyte cell hemoglobin content; CHr†, reticulocyte hemoglobin content; RetHb, reticulocyte hemoglobin. (Abbreviation expansions are presented as described in the original studies. Superscripts were added where the same abbreviation was used with different term expansions.).
(DOCX)

S12 Table Changes in Ret-He/CHr before and after iron therapy in observational studies. Observational studies reporting changes in Ret-He/CHr before and after iron therapy during pregnancy or the postpartum period are summarized. Data are presented as mean ± standard deviation as reported in the original studies. *N* indicates the total number of women included in each study; subgroup-specific sample sizes are shown where applicable. *Before* and *After* represent Ret-He/CHr values measured at baseline and after completion of iron therapy, respectively. *P* values correspond to within-group comparisons, as reported in the original studies. In Ramakers et al. [26], postpartum hemorrhage was defined as blood loss > 500 mL. T, timing of treatment or intervention initiation. IDE*, iron-deficient erythropoiesis (Hb ≤ 11.0 g/dL, MCV 80–100 fL, Ret-He < 29.6 pg and ZPP > 75 μmol/mol heme). IDE**, iron-deficient erythropoiesis (Hb ≤ 11.0 g/dL, MCV 80–100 fL, Ret-He < 29.8 pg, delta-He < 0 or ZPP > 75 μmol/mol heme). qd, once daily; bid, twice daily; tid, three times a day. Abbreviations: CHr, reticulocyte hemoglobin content; Ret-He, hemoglobin content of reticulocytes; Ret-Hb, reticulocyte-hemoglobin; Hb, hemoglobin; MCV, mean corpuscular volume; ZPP, zinc protoporphyrin heme ratio; delta-He, Ret-He minus RBC-He; TSAT, transferrin saturation. (Abbreviation expansions are presented as described in the original studies.).
(DOCX)

S13 Table Changes in Ret-He/CHr value before and after iron-based interventions. Studies reporting changes in Ret-He/CHr before and after iron-based interventions during pregnancy or the postpartum period are summarized. Data are presented as mean ± standard deviation or median (interquartile range), as reported in the original studies. *N* indicates the total number of women included in each study; comparison group–specific sample sizes are shown where applicable. *T* indicates the timing of treatment or intervention initiation. *Before* and *After* represent Ret-He/CHr values measured at baseline and after completion of the intervention, respectively. *P* values correspond to within-group comparisons, as reported in the original studies. **A. Changes in MCHr before and after iron-based intervention in pregnant women with β-thalassemia trait [19].** Abbreviations: RhEPO, recombinant human erythropoietin; i.v., intravenous; MCHr, mean reticulocyte hemoglobin content. **B. Changes in MCHr before and after postpartum iron supplementation in women with antenatal non-anemic iron deficiency [22].** Abbreviations: MCHr, term expansion not specified in the original study; ID, iron deficiency; Hb, hemoglobin. **C. Changes in CHr before and after iron-based treatment in pregnant women with moderate anemia [31].** IDA, iron deficiency anemia, defined as HRC > 2.5%, CHr < 28 pg, and RDW > 15%. ACD, anemia of chronic disorders, defined in the original study as HRC < 2.5%, CHr > 28 pg, RDW < 15%, EPO < 50 U/L, and Hb < 10 g/dL. Abbreviations: RhEPO, recombinant human erythropoietin; i.v., intravenous; CHr, reticulocyte hemoglobin content; Hb, hemoglobin; HRC, hypochromic red blood cell percentage; RDW, red blood cell distribution width. **D. Changes in RET-He before and after iron-based intervention with or without adjunctive supplementation in pregnant women with mild to moderate anemia [48].** Abbreviations: bid, twice daily; Hb, hemoglobin; RET-He, reticulocyte hemoglobin equivalent. **E. Changes in RET-He before and after iron-based intervention in pregnant women with iron-deficiency anemia [52].** Abbreviations: Hb, hemoglobin; RET-He, reticulocyte hemoglobin.
(DOCX)

S14 Checklist PRISMA-ScR checklist. (PDF)
