# Supplementary material for: Clinical applications of reticulocyte hemoglobin indices, including reticulocyte hemoglobin equivalent (Ret-He) and reticulocyte hemoglobin content (CHr), among pregnant and postpartum women: A scoping review and evidence map
Source: PLoS One. 2026 Nov 13;21(11):e0362481. doi: 10.1371/journal.pone.0362481 (PMC13766218; doi:10.1371/journal.pone.0362481)
Supplement: Supplementary file 2 [file pone.0362481.s001.docx]

**S1 Appendix. Full search strategies**

**A. Full search strategy for Pubmed**

***Original search***

Date searched: June 4, 2025

Search strategy:

(

(

Ret-He[tiab] OR RetHe[tiab]

OR (CHr[tiab] AND Reticulocyte[tiab])

OR (RHe[tiab] AND Reticulocyte[tiab])

OR "Reticulocyte Hemoglobin"[tiab]

OR "Reticulocyte Haemoglobin"[tiab]

OR "Reticulocyte Hb"[tiab]

OR "Reticulocytes"[Mesh]

)

AND

(

Pregnanc*[tiab] OR Pregnant[tiab] OR Gestation*[tiab]

OR Antenatal[tiab] OR Prenatal[tiab] OR Perinatal[tiab]

OR Postpartum[tiab] OR Gravidity[tiab] OR Maternal[tiab]

OR Partum[tiab] OR Parturition*[tiab] OR obstetric*[tiab]

OR "Pregnancy"[Mesh] OR "Pregnant People"[Mesh]

OR "Prenatal Care"[Mesh] OR "Postpartum Period"[Mesh]

OR "Obstetrics"[Mesh]

)

)

AND("1990/01/01"[dp] : "2025/05/31"[dp])

***Updated search***

Date searched: April 21, 2026

Search strategy:

(

(

Ret-He[tiab] OR RetHe[tiab]

OR (CHr[tiab] AND Reticulocyte[tiab])

OR (RHe[tiab] AND Reticulocyte[tiab])

OR "Reticulocyte Hemoglobin"[tiab]

OR "Reticulocyte Haemoglobin"[tiab]

OR "Reticulocyte Hb"[tiab]

OR "Reticulocytes"[Mesh]

)

AND

(

Pregnanc*[tiab] OR Pregnant[tiab] OR Gestation*[tiab]

OR Antenatal[tiab] OR Prenatal[tiab] OR Perinatal[tiab]

OR Postpartum[tiab] OR Gravidity[tiab] OR Maternal[tiab]

OR Partum[tiab] OR Parturition*[tiab] OR obstetric*[tiab]

OR "Pregnancy"[Mesh] OR "Pregnant People"[Mesh]

OR "Prenatal Care"[Mesh] OR "Postpartum Period"[Mesh]

OR "Obstetrics"[Mesh]

)

)

AND ("2025/06/01"[crdt] : "2026/04/21"[crdt])

**B. Full search strategy for CENTRAL (Cochrane Central Register of Controlled Trials)**

***Original search***

Date searched: June 1, 2025

Search strategy:

#1

(Ret-He OR RetHe OR (CHr AND Reticulocyte) OR (RHe AND Reticulocyte)

OR "Reticulocyte Hb" OR "Reticulocyte Haemoglobin" OR "Reticulocyte Hemoglobin"):ti,ab,kw

#2

[mh Reticulocytes]

#3

(Pregnanc* OR Pregnant OR Gestation* OR Antenatal OR Prenatal OR Perinatal OR Postpartum OR Gravidity OR Maternal OR Partum OR Parturition* OR Obstetric):ti,ab,kw

#4

[mh Pregnancy] OR [mh "Pregnant People"] OR [mh "Prenatal Care"] OR [mh Postpartum Period] OR [mh Obstetrics]

#5

(#1 OR #2) AND (#3 OR #4)

Interface limits:

Year first published: 1990 to 2025

Note:

The original CENTRAL search was limited by year first published (1990–2025). The results were checked for records first published after May 31, 2025, and no records outside the prespecified publication-date range were identified.

***Updated search***

Date searched: April 21, 2026

Search strategy:

#1–#5: Same as original search

Interface limits:

Date added to CENTRAL trials database: June 1, 2025 to April 21, 2026

**C. Full search strategy for Web of Science**

***Original search***

Date searched: June 4, 2025

Search strategy:

#1

ALL=("RET-He" OR "Reticulocyte*" OR "RetHe")

#2

ALL=(Pregnanc* OR Pregnant OR Gestation* OR Antenatal OR Prenatal OR Perinatal OR Postpartum OR Gravidity OR Maternal OR Partum OR Parturition* OR Obstetric)

#3

#1 AND #2

Interface limits:

Publication date range: January 1, 1990 to May 31, 2025

***Updated search***

Date searched: April 21, 2026

Search strategy:

#1–#3: Same as original search

Interface limits:

Index date range: June 1, 2025 to April 21, 2026

**D. Full search strategy for Ichushi (Japan Medical Abstracts Society)**

English translations of the Japanese-language search terms used in this section and Section E are provided in Section F. The original search expressions have been retained to preserve reproducibility.

***Original search***

Date searched: June 4, 2025

Search strategy:

("網状赤血球"/AL or Ret-He/AL or Reticulocyte/AL or 網赤血球/AL

or "網状赤血球"/TH or "網状赤血球計数"/TH)

and

(妊娠/TA or 妊婦/TA or 妊産婦/TA or 周産期/TA or 分娩/TA or Pregnancy/TA

or Pregnant/TA or Gestation/TA or Prenatal/TA or Postpartum/TA or Maternal/TA

or 産褥/TA or 産後/TA or 母体/TA or 出産/TA or Antenatal/TA or Puerperium/TA

or "妊娠"/TH or "妊娠管理"/TH or "妊娠期"/TH or "妊産婦"/TH or "妊娠合併症"/TH

or "周産期"/TH or "産褥"/TH or "産後管理"/TH)

Interface limits:

Publication date range: January 1, 1990 to May 31, 2025

***Updated search***

Date searched: April 21, 2026

Search strategy:

Same as original search

Interface limits:

Index date range: June 1, 2025 to April 21, 2026

**E. Supplementary validation search for additional analyzer-specific terminology**

**Database: PubMed**
Date searched: June 13, 2026
Search strategy:
(
(
"reticulocyte haemoglobin cellular content"[tiab]
OR "reticulocyte hemoglobin cellular content"[tiab]
OR "reticulocyte hemoglobin expression"[tiab]
OR "reticulocyte haemoglobin expression"[tiab]
OR (RHE[tiab] AND reticulocyte*[tiab])
OR (RHCc[tiab] AND reticulocyte*[tiab])
)
AND
(
Pregnanc*[tiab] OR Pregnant[tiab] OR Gestation*[tiab]
OR Antenatal[tiab] OR Prenatal[tiab] OR Perinatal[tiab]
OR Postpartum[tiab] OR Gravidity[tiab] OR Maternal[tiab]
OR Partum[tiab] OR Parturition*[tiab] OR obstetric*[tiab]
OR "Pregnancy"[Mesh] OR "Pregnant People"[Mesh]
OR "Prenatal Care"[Mesh] OR "Postpartum Period"[Mesh]
OR "Obstetrics"[Mesh]
)
)
AND ("1990/01/01"[dp] : "2026/06/13"[dp])

Records identified: 1
Additional eligible studies: 0; the retrieved record had already been included in the review.

**Database: CENTRAL**
Date searched: June 13, 2026
Search strategy:
#1
("reticulocyte haemoglobin cellular content"
OR "reticulocyte hemoglobin cellular content"
OR "reticulocyte hemoglobin expression"
OR "reticulocyte haemoglobin expression"
OR (RHE AND reticulocyte*)
OR (RHCc AND reticulocyte*)):ti,ab,kw

#2
(Pregnanc* OR Pregnant OR Gestation* OR Antenatal OR Prenatal OR Perinatal OR Postpartum OR Gravidity OR Maternal OR Partum OR Parturition* OR Obstetric):ti,ab,kw

#3
[mh Pregnancy] OR [mh "Pregnant People"] OR [mh "Prenatal Care"] OR [mh Postpartum Period] OR [mh Obstetrics]

#4
#1 AND (#2 OR #3)

Records identified: 0
Additional eligible studies: 0

**Database: Web of Science**
No separate supplementary validation search was performed because the original and updated Web of Science strategies already included the broader term “Reticulocyte*” in the ALL field.

**Database: Ichushi Web**
Date searched: June 13, 2026
Search strategy:
("reticulocyte haemoglobin cellular content"/AL
OR "reticulocyte hemoglobin cellular content"/AL
OR "reticulocyte hemoglobin expression"/AL
OR "reticulocyte haemoglobin expression"/AL)
and
(妊娠/TA or 妊婦/TA or 妊産婦/TA or 周産期/TA or 分娩/TA or Pregnancy/TA
or Pregnant/TA or Gestation/TA or Prenatal/TA or Postpartum/TA or Maternal/TA
or 産褥/TA or 産後/TA or 母体/TA or 出産/TA or Antenatal/TA or Puerperium/TA
or "妊娠"/TH or "妊娠管理"/TH or "妊娠期"/TH or "妊産婦"/TH or "妊娠合併症"/TH
or "周産期"/TH or "産褥"/TH or "産後管理"/TH)

Records identified: 0
Additional eligible studies: 0

**F. English translations of Japanese-language search terms used in the Ichushi Web search strategies**

The original Japanese search terms and field tags were retained in Sections D and E to ensure reproducibility of the searches. English translations of all Japanese-language search terms are provided below for clarity. These translations are explanatory only and were not used as substitutes for the original search terms.

| **Japanese search term** | **English translation** |
| --- | --- |
| 網状赤血球 | Reticulocyte |
| 網赤血球 | Reticulocyte |
| 網状赤血球計数 | Reticulocyte count |
| 妊娠 | Pregnancy |
| 妊婦 | Pregnant women |
| 妊産婦 | Pregnant and postpartum women |
| 周産期 | Perinatal period |
| 分娩 | Labor and delivery |
| 産褥 | Puerperium |
| 産後 | Postpartum period |
| 母体 | Maternal |
| 出産 | Childbirth |
| 妊娠管理 | Pregnancy management |
| 妊娠期 | Gestational period |
| 妊娠合併症 | Pregnancy complications |
| 産後管理 | Postpartum care |
