# Supplementary material for: Clinical applications of reticulocyte hemoglobin indices, including reticulocyte hemoglobin equivalent (Ret-He) and reticulocyte hemoglobin content (CHr), among pregnant and postpartum women: A scoping review and evidence map
Source: PLoS One. 2026 Nov 13;21(11):e0362481. doi: 10.1371/journal.pone.0362481 (PMC13766218; doi:10.1371/journal.pone.0362481)
Supplement: Supplementary file 3 [file pone.0362481.s002.docx]

**S2 Table. Studies excluded at full-text screening stage and reasons for exclusion**

| Author (year) | Reason for exclusion |
| --- | --- |
| Abhilashini GD (2014) | Ret-He/CHr not measured or evaluated |
| Bayoumeu F (2002) | Ret-He/CHr not measured or evaluated |
| Bhargava R (2013) | Ret-He/CHr not measured or evaluated |
| Bresani CC (2013) | Ret-He/CHr not measured or evaluated |
| Breymann C (1995) | Ret-He/CHr not measured or evaluated |
| Breymann C (1996) | Ret-He/CHr not measured or evaluated |
| Breymann C (2000) | Ret-He/CHr not measured or evaluated |
| Breymann C (2001) | Ret-He/CHr not measured or evaluated |
| Bumrungpert A (2022) | Ret-He/CHr not measured or evaluated |
| Choi JW (2001) | Ret-He/CHr not measured or evaluated |
| de Freitas MAR (2018) | Ret-He/CHr not measured or evaluated |
| Dennis K (2022) | Ret-He/CHr not measured or evaluated |
| Dursun ZE (2023) | Ret-He/CHr not measured or evaluated |
| Ervasti M (2009a) | Overlapping population with an included study |
| Ervasti M (2009b) | Overlapping population with an included study |
| Gosavi M (2021) | Ret-He/CHr not measured or evaluated |
| Haizler-Cohen L (2024) | Duplicate publication |
| Hennek JW (2016) | Analytical/laboratory validation only |
| Huch A (1992) | Ret-He/CHr not measured or evaluated |
| Hye RA (2022) | Ret-He/CHr not measured or evaluated |
| Idi HT (2019) | Ret-He/CHr not measured or evaluated |
| John NM (2026) | Population not pregnant or postpartum |
| Kanwal A (2021) | Ret-He/CHr not measured or evaluated |
| Kitaoka H (2023) | Population not pregnant or postpartum |
| Kochhar PK (2013) | Ret-He/CHr not measured or evaluated |
| Kriplani A (2013) | Ret-He/CHr not measured or evaluated |
| Lao TT (2002) | Ret-He/CHr not measured or evaluated |
| Lao TT (2004) | Ret-He/CHr not measured or evaluated |
| Lee R (2025) | Ret-He/CHr not measured or evaluated |
| Mahdi AH (2023) | Population not pregnant or postpartum |
| Makrydimas G (1998) | Ret-He/CHr not measured or evaluated |
| Manu B (2026) | Review article |
| Martens KL (2026) | Review article |
| Nishijima M (2022) | Ret-He/CHr not measured or evaluated |
| Oseni BSA (2010) | Ret-He/CHr not measured or evaluated |
| Rao RB (2024) | Review article |
| Ronchi F (2021) | Population not pregnant or postpartum |
| Shimizu T (2016) | Ret-He/CHr not measured or evaluated |
| Skikne B (2012) | Review article |
| Takahashi Y (2022) | Ret-He/CHr not measured or evaluated |
| Takeuchi M (2016) | Ret-He/CHr not measured or evaluated |
| Uma G (2013) | Ret-He/CHr not measured or evaluated |
| Vega-Sánchez R (2020) | Ret-He/CHr not measured or evaluated |
| Wagh G (2019) | Ret-He/CHr not measured or evaluated |
| Xiao HJ (2021) | Ret-He/CHr not measured or evaluated |
| Yokouchi T (2015) | Population not pregnant or postpartum |
| Younis MS (2017) | Ret-He/CHr not measured or evaluated |
| Zhang HD (2020) | Population not pregnant or postpartum |
| Zupanic D (2025) | Ret-He/CHr not measured or evaluated |
