# Supplementary material for: Clinical applications of reticulocyte hemoglobin indices, including reticulocyte hemoglobin equivalent (Ret-He) and reticulocyte hemoglobin content (CHr), among pregnant and postpartum women: A scoping review and evidence map
Source: PLoS One. 2026 Nov 13;21(11):e0362481. doi: 10.1371/journal.pone.0362481 (PMC13766218; doi:10.1371/journal.pone.0362481)
Supplement: Supplementary file 4 [file pone.0362481.s003.docx]

S3 Table. Diagnostic performance of Ret-He/CHr for iron deficiency anemia or microcytic anemia

| Author (year) | Country | N | T | Parameter | Analyzers | Definition of anemia | Cut-off (pg) | AUC ±SE or (95%CI) | Sensitivity / specificity | AUC of comparator indices |
| --- | --- | --- | --- | --- | --- | --- | --- | --- | --- | --- |
| Ervasti (2007) [23] | Finland | 202 | admission for delivery | CHr | Advia 120 | Hb ≤12.2 g/dL, MCV ≤86 fL, and ferritin ≤11 μg/L | 30.5 | 0.95 ± 0.03 | 92.3 / 88.7 | sTfR: 0.79 ± 0.06 |
| Ervasti (2007) [23] | Finland | 202 | admission for delivery | CHr | Advia 120 | Hb ≤12.2 g/dL and MCV ≤86 fL | ·· | 0.82 ± 0.06 | ·· | sTfR: 0.74 ± 0.05 ferritin: 0.75 ± 0.05 |
| Priddee (2020) [37] | United Kingdom | 1966 | first trimester | RET-He | ·· | microcytic or normocytic anaemia | ·· | 0.817 | ·· | ferritin: 0.667 |
| Haizler-Cohen (2025) [49] | United States | 196 | antenatal (unspecified) | Ret-He | ·· | Hb <11 g/dL (second trimester: <10.5 g/dL) and ferritin <30 μg/L | ·· | 0.82 (0.75-0.89) | ·· | ·· |
