# Supplementary material for: Clinical applications of reticulocyte hemoglobin indices, including reticulocyte hemoglobin equivalent (Ret-He) and reticulocyte hemoglobin content (CHr), among pregnant and postpartum women: A scoping review and evidence map
Source: PLoS One. 2026 Nov 13;21(11):e0362481. doi: 10.1371/journal.pone.0362481 (PMC13766218; doi:10.1371/journal.pone.0362481)
Supplement: Supplementary file 5 [file pone.0362481.s004.docx]

S4 Table. Diagnostic performance of Ret-He/CHr for iron deficiency (ID) defined by serum ferritin

| Author (year) | Country | N | T | Parameter | Analyzers | Ferritin threshold for ID | Subgroup (secondary analyses) | Cut-off (pg) | AUC (95%CI) | Sensitivity / specificity | AUC of comparator indices |
| --- | --- | --- | --- | --- | --- | --- | --- | --- | --- | --- | --- |
| Priddee (2020) [37] | United Kingdom | 1966 | first trimester | RET-He | ·· | <30 μg/L | ·· | ·· | 0.72 | ·· | Hb: 0.70 |
| Bó (2023) [42] | Brazil | 60 | antenatal (unspecified) | RET-He | Sysmex XN10 Roche | <13μg/L | ·· | ·· | 0.73 (0.57-0.89) | ·· | Hb: 0.59 (0.39-0.78) MCHC: 0.55 (0.35-0.76) MCV: 0.48 (0.31-0.66) |
| Sahithya (2024) [46] | India | 280 | first trimester | Ret-He† | Sysmex XN 1000 | <20 μg/L | ·· | 32.25 | 0.99 (0.99-1.00) | 98.0 / 97.0 | ·· |
| Shu (2024) [47] | China | 724 | antenatal (unspecified) | RHE | Mindray BC-6900 | <15 μg/L | ·· | 29.85 | 0.84 (0.81-0.87) | 87.1 / 66.3 | MCV: 0.65 (0.59-0.68) MCH: 0.63 (0.61-0.64) MCHC: 0.60 (0.55-0.64) |
| Haizler-Cohen (2025) [49] | United States | 196 | antenatal (unspecified) | Ret-He | ·· | <30 μg/L | overall (n = 196) | 34.0 | 0.65 (0.55-0.75) | 72.1 / 53.9 | ·· |
| Haizler-Cohen (2025) [49] | United States | 196 | antenatal (unspecified) | Ret-He | ·· | <30 μg/L | not treated with iron (n = 179) | ·· | 0.66 (0.56-0.76) | ·· | ·· |
| Haizler-Cohen (2025) [49] | United States | 196 | first trimester | Ret-He | ·· | <30 μg/L | first trimester (n = 51) | ·· | 0.57 (0.41-0.73) | ·· | ·· |
| Haizler-Cohen (2025) [49] | United States | 196 | second trimester | Ret-He | ·· | <30 μg/L | second trimester (n = 68) | ·· | 0.64 (0.43-0.84) | ·· | ·· |
| Haizler-Cohen (2025) [49] | United States | 196 | third trimester | Ret-He | ·· | <30 μg/L | third trimester (n = 77) | ·· | 0.53 (0.15-0.91) | ·· | ·· |
| Haizler-Cohen (2025) [49] | United States | 196 | antenatal (unspecified) | Ret-He | ·· | <15 μg/L | overall (n = 196) | ·· | 0.72 (0.65-0.79) | ·· | ·· |
