# Supplementary material for: Clinical applications of reticulocyte hemoglobin indices, including reticulocyte hemoglobin equivalent (Ret-He) and reticulocyte hemoglobin content (CHr), among pregnant and postpartum women: A scoping review and evidence map
Source: PLoS One. 2026 Nov 13;21(11):e0362481. doi: 10.1371/journal.pone.0362481 (PMC13766218; doi:10.1371/journal.pone.0362481)
Supplement: Supplementary file 6 [file pone.0362481.s005.docx]

S5 Table. Diagnostic performance of Ret-He/CHr for iron deficiency (ID) defined by transferrin saturation (TSAT)

| Author (year) | Country | N | T | Parameter | Analyzers | TSAT threshold for ID | Subgroup (secondary analyses) | Cut-off (pg) | AUC (95%CI) | Sensitivity / specificity | AUC of comparator indices |
| --- | --- | --- | --- | --- | --- | --- | --- | --- | --- | --- | --- |
| Ervasti (2007) [23] | Finland | 202 | admission for delivery | CHr | Advia 120 | ≤11% | ·· | 31.9 | 0.79 (0.71-0.87) | 80.7 / 71.3 | Hb: 0.64 (0.54-0.74)  MCV: 0.63 (0.53-0.73) ferritin: 0.77 (0.68-0.85) sTfR: 0.67 (0.57-0.77) |
| Urrechaga (2014) [28] | Spain | 70 | 20 weeks’ gestation | RetHe | Sysmex XE500 | <15% | ·· | 29.9 | 0.79 (0.70-0.88) | 80.7 / 71.3 | Hb: 0.69 (0.59-0.79) MCH: 0.71 (0.61-0.81) MCV: 0.69 (0.59-0.79) |
| Casper (2024) [45] | United States | 165 | ≤14 weeks’ gestation | RET-He | ·· | <20% | overall (n = 165) | 33.2 | 0.77 (0.67-0.88) | 63 / 91 | ferritin: 0.64 (0.52-0.75) |
| Casper (2024) [45] | United States | 165 | ≤14 weeks’ gestation | RET-He | ·· | <20% | BMI ≥30 (n = 82) | 33.0 | 0.76 (0.64-0.88) | 59 / 92 | ferritin: 0.60 (0.45-0.74) |
| Casper (2024) [45] | United States | 165 | ≤14 weeks’ gestation | RET-He | ·· | <20% | BMI <30 (n = 83) | 32.8 | 0.73 (0.49-0.96) | 56 / 96 | ferritin: 0.76 (0.60-0.93) |
