# Supplementary material for: Clinical applications of reticulocyte hemoglobin indices, including reticulocyte hemoglobin equivalent (Ret-He) and reticulocyte hemoglobin content (CHr), among pregnant and postpartum women: A scoping review and evidence map
Source: PLoS One. 2026 Nov 13;21(11):e0362481. doi: 10.1371/journal.pone.0362481 (PMC13766218; doi:10.1371/journal.pone.0362481)
Supplement: Supplementary file 7 [file pone.0362481.s006.docx]

S6 Table. Diagnostic performance of Ret-He/CHr for iron deficiency (ID) defined using composite reference standards

| Author (year) | Country | N | T | Parameter | Analyzers | Composite definition of ID | Cut-off (pg) | AUC (95%CI) | Sensitivity / specificity | AUC of comparator indices |
| --- | --- | --- | --- | --- | --- | --- | --- | --- | --- | --- |
| Levy (2018) [32] | South Africa | 102 | first trimester | Ret-He | Sysmex XN 9000 | serum iron <9 μmol/L, TSAT <20% ,or ferritin <30 μg/L | 31.2 | 0.81 (0.71–0.88) | 62.5 / 86.4 | Hb: 0.66 (0.56-0.75) MCV: 0.76 (0.65-0.83) MCH: 0.78 (0.69-0.86) |
| Kumar (2020) [35] | India | 155 | first trimester | Ret-He | Sysmex XN 1000 | MCV <83 fL and MCH <27 pg | 27.8 | 0.93 (0.90–0.98) | 93 / 83 | ferritin: 0.76 (0.66-0.87) |
