# Supplementary material for: Clinical applications of reticulocyte hemoglobin indices, including reticulocyte hemoglobin equivalent (Ret-He) and reticulocyte hemoglobin content (CHr), among pregnant and postpartum women: A scoping review and evidence map
Source: PLoS One. 2026 Nov 13;21(11):e0362481. doi: 10.1371/journal.pone.0362481 (PMC13766218; doi:10.1371/journal.pone.0362481)
Supplement: Supplementary file 8 [file pone.0362481.s007.docx]

S7 Table. Associations between Ret-He/CHr and ferritin

| Author (year) | Country | N | T | Parameter | Analyzers | Statistical analysis | Results |
| --- | --- | --- | --- | --- | --- | --- | --- |
| Krafft (2003) [21] | Switzerland | 64 | postpartum | CHr | Advia | spearman correlation | r =0 .42, p <0.01 |
| Bjørke-Monsen (2012) [25] | Norway | 104 | postpartum | CHr | Advia 120 | generalized additive models (GAM) | nonlinear trend; inflection at ferritin around 20 μg/L |
| Demmers (2019) [33] | Netherlands | 926 | first trimester | MCHr | CELL-DYN Sapphire | logistic regression analysis | significantly associated with low ferritin (<20 μg/L, p ＜0.001) |
| Kumar (2022) [39] | India | 155 | first trimester | Ret-He | Sysmex-XN 1000 | spearman correlation | r= 0.5, p<0.001 |
| Santana (2022) [40] | United States | 37 | antenatal (unspecified) | RET-He | ·· | spearman correlation | r = 0.24, p = 0.15 |
| Haizler-Cohen (2025) [49] | United States | 196 | antenatal (unspecified) | Ret-He | ·· | spearman correlation | rs = 0.48, p <0.0001 |
