# Supplementary material for: Clinical applications of reticulocyte hemoglobin indices, including reticulocyte hemoglobin equivalent (Ret-He) and reticulocyte hemoglobin content (CHr), among pregnant and postpartum women: A scoping review and evidence map
Source: PLoS One. 2026 Nov 13;21(11):e0362481. doi: 10.1371/journal.pone.0362481 (PMC13766218; doi:10.1371/journal.pone.0362481)
Supplement: Supplementary file 9 [file pone.0362481.s008.docx]

S8 Table. Associations between Ret-He/CHr and other iron-related markers, erythrocyte indices, or inflammatory markers

|  | Country | N | T | Parameter | Analyzers | Correlation coefficient (r, p value) | | |
| --- | --- | --- | --- | --- | --- | --- | --- | --- |
| Author (year) |  |  |  |  |  | Irom-related markers | Erythrocyte indices | Inflammatory |
| Krafft (2003) [21] | Switzerland | 64 | postpartum | CHr | Advia | sTfR: -0.53 (<0.01) TSAT: 0.13 | Hb: 0.35  HRC: -0.61 (<0.01) | CRP: -0.13  neopterin: -0.09 |
| Ervasti (2008) [24] | Finland | 210 | admission for delivery | CHr**†** | Advia 120 | EPO: -0.45 | ·· | ·· |
| Bjørke-Monsen (2012) [25] | Norway | 104 | postpartum | CHr | Advia 120 | ·· | ·· | neopterin: -0.25 (0.06) |
| Ramakers (2012) [26] | Netherlands | 195 | postpartum | CHr | Advia 120 | ·· | MCV: 0.66 (R2, <0.001) | ·· |
| Santana (2022) [40] | United States | 37 | antenatal (unspecified) | RET-He | ·· | ·· | MCV: 0.86 (<0.0001) | ·· |
| Bó (2023) [42] | Brazil | 60 | antenatal (unspecified) | RET-He**†** | Sysmex XN10 Roche | ·· | IRF: -0.30 (0.022) | ·· |
| Sahithya (2024) [46] | India | 280 | first trimester | Ret-He | Sysmex XN 1000 | ·· | MCV: 0.39 (<0.001) | ·· |
