# Supplementary material for: Clinical applications of reticulocyte hemoglobin indices, including reticulocyte hemoglobin equivalent (Ret-He) and reticulocyte hemoglobin content (CHr), among pregnant and postpartum women: A scoping review and evidence map
Source: PLoS One. 2026 Nov 13;21(11):e0362481. doi: 10.1371/journal.pone.0362481 (PMC13766218; doi:10.1371/journal.pone.0362481)
Supplement: Supplementary file 10 [file pone.0362481.s009.docx]

S9 Tables. Associations between maternal Ret-He/CHr and neonatal parameters

A. Correlations between maternal and neonatal Ret-He/CHr

| Author (year) | Country | N | T | Parameter | Analyzers | Subgroup (secondary analyses) | Correlation coefficient (r, p value) |
| --- | --- | --- | --- | --- | --- | --- | --- |
| Babacheva (2022) [38] | Greece | 200 | postpartum | MCHr | Alinity Hq | ·· | 0.147 (0.112) |
| Casper (2025) [50] | United States | 65 | admission for delivery | RET-He | ·· | overall (n = 65) | 0.323 (0.009) |
| Casper (2025) [50] | United States | 65 | admission for delivery | RET-He | ·· | BMI ≥30 (n = 27) | 0.438 (0.024) |
| Casper (2025) [50] | United States | 65 | admission for delivery | RET-He | ·· | BMI <30 (n = 38) | 0.278 (0.091) |

B. Predictive performance of maternal RET-He for neonatal anemia and low neonatal RET-He [53]

| Author (year) | Country | N | T | Parameter | Analyzers | Diagnostic target | Cut-off (pg) | AUC (95%CI) | Sensitivity / specificity |
| --- | --- | --- | --- | --- | --- | --- | --- | --- | --- |
| Pekar (2025) [53] | United States | 177 | first trimester | RET-He | Sysmex XN | Neonatal HgB<13.5g/dL | ·· | 0.54 (0.40-0.68) | 11/76 |
| Pekar (2025) [53] | United States | 177 | third trimester | RET-He | Sysmex XN | Neonatal HgB<13.5g/dL | ·· | 0.51 (0.35-0.66) | 44/49 |
| Pekar (2025) [53] | United States | 177 | first trimester | RET-He | Sysmex XN | Neonatal RET-He < 29 pg | ·· | 0.89 (0.79-0.98) | 83/80 |
| Pekar (2025) [53] | United States | 177 | third trimester | RET-He | Sysmex XN | Neonatal RET-He < 29 pg | ·· | 0.82 (0.70-0.94) | 100/16 |
