# Supplementary material for: Clinical applications of reticulocyte hemoglobin indices, including reticulocyte hemoglobin equivalent (Ret-He) and reticulocyte hemoglobin content (CHr), among pregnant and postpartum women: A scoping review and evidence map
Source: PLoS One. 2026 Nov 13;21(11):e0362481. doi: 10.1371/journal.pone.0362481 (PMC13766218; doi:10.1371/journal.pone.0362481)
Supplement: Supplementary file 11 [file pone.0362481.s010.docx]

S10 Table. Reference range studies of Ret-He/CHr during pregnancy and the postpartum period

| Author (year) | Country | N | Participants | Parameter | Analyzers | Summary statistics | Stratification / Exclusion | First trimester | Second trimester | Third trimester | Postpartum |
| --- | --- | --- | --- | --- | --- | --- | --- | --- | --- | --- | --- |
| Yu (2019) [34] | China | 133 | non-anemic and singleton primiparas | Ret-He | Sysmex XE-2100 | 95%RV | no | 29.8-39.6 | 29.2-40.8 | 26.2-41.0 | 26.8-38.5 |
| Kumar (2022) [39] | India | 59 | Hb>11g/dL, MCV83-98fL, and ferritin>15ng/mL | Ret-He**†** | Sysmex-XN 1000 | median (90%CI / IQR) | exclusion (ID, iron supplementation) | 29 (29.5-34.2 / 31.8-32.8) | ·· | ·· | ·· |
