# Supplementary material for: Clinical applications of reticulocyte hemoglobin indices, including reticulocyte hemoglobin equivalent (Ret-He) and reticulocyte hemoglobin content (CHr), among pregnant and postpartum women: A scoping review and evidence map
Source: PLoS One. 2026 Nov 13;21(11):e0362481. doi: 10.1371/journal.pone.0362481 (PMC13766218; doi:10.1371/journal.pone.0362481)
Supplement: Supplementary file 12 [file pone.0362481.s011.docx]

S11 Table. Longitudinal changes in Ret-He/CHr during pregnancy and postpartum

| Author (year) | Country | N | Participants | Parameter | Analyzers | Summary statistics | Stratification / Exclusion | First trimester | Second trimester | Postpartum (timing) |
| --- | --- | --- | --- | --- | --- | --- | --- | --- | --- | --- |
| Richter (1999) [20] | Switzerland | 82 | puerperal women (uncomplicated) | CHr | Technicon H3 RTC | mean | not reported | ·· | ·· | 38.0 (day 0) 30.2 (day 2) 30.2 (day 42) |
| Bjørke-Monsen (2012) [25] | Norway | 104 | healthy puerperal women | CHr**†** | Advia 120 | median (IQR) | no | ·· | ·· | wk 6: 32.1 (30.7-33.0) mo 4: 32.3 (31.2-33.3) mo 11: 33.1 (31.9-34.0) |
| Griffin (2023) [43] | United States | 499 | non-anemic (first trimester) and singleton women | RetHb | ·· | mean ±SD | stratification (ID) | overall: 34.3 ± 2.2 | overall: 32.9 ± 3.0 | ·· |
| Griffin (2023) [43] | United States | 499 | non-anemic (first trimester) and singleton women | RetHb | ·· | mean ±SD | stratification (ID) | ID (n = 76): 33.4 ± 2.4 | ID (n = 349): 32.2 ± 3.2 | ·· |
| Griffin (2023) [43] | United States | 499 | non-anemic (first trimester) and singleton women | RetHb | ·· | mean ±SD | stratification (ID) | non-ID (n = 423): 34.5 ± 1.9 | non-ID (n = 150): 34.0 ± 2.6 | ·· |
