# Supplementary material for: Clinical applications of reticulocyte hemoglobin indices, including reticulocyte hemoglobin equivalent (Ret-He) and reticulocyte hemoglobin content (CHr), among pregnant and postpartum women: A scoping review and evidence map
Source: PLoS One. 2026 Nov 13;21(11):e0362481. doi: 10.1371/journal.pone.0362481 (PMC13766218; doi:10.1371/journal.pone.0362481)
Supplement: Supplementary file 13 [file pone.0362481.s012.docx]

S12 Table. Changes in Ret-He/CHr before and after iron therapy in observational studies

| Author(year) | Country | N | T | Participant | Parameter | Analyzers | Treatment | Subgroup definition | Before (pg) | After (pg) | P value |
| --- | --- | --- | --- | --- | --- | --- | --- | --- | --- | --- | --- |
| Ramakers (2012) [26] | Netherlands | 195 | postpartum (week 0) | postpartum hemorrhage or clinical anemia | CHr | Advia 120 | ferrous fumarate 200 mg tid for 4 weeks | anemia (Hb:6.5-10.4, n=105) | 29.8 ± 2.9 | 29.8 ± 2.6 | ·· |
| Ramakers (2012) [26] | Netherlands | 195 | postpartum (week 0) | postpartum hemorrhage or clinical anemia | CHr | Advia 120 | ferrous fumarate 200 mg tid for 4 weeks | anemia (baseline CHr<28 pg, n=30) | 26.1 ± 1.4 | 28.0 ± 2.6 | <0.001 |
| Ramakers (2012) [26] | Netherlands | 195 | postpartum (week 0) | postpartum hemorrhage or clinical anemia | CHr | Advia 120 | ferrous fumarate 200 mg tid for 4 weeks | anemia (baseline CHr≥28 pg, n=75) | 31.4 ± 1.8 | 30.4 ± 2.3 | <0.001 |
| Ramakers (2012) [26] | Netherlands | 195 | postpartum (week 0) | postpartum hemorrhage or clinical anemia | CHr | Advia 120 | observation | non-anemia (Hb≥10.5, n=90) | 31.6 ± 2.4 | 30.8 ± 2.1 | <0.001 |
| Schoorl (2012) [27] | Netherlands | 25 | third trimester | suspected IDE * | Ret-He | Sysmex XE2100 | ferrous fumarate 200 mg bid (200 mg Fe/day), 4 weeks | ·· | 23.6 ± 2.8 | 28.3 ± 2.6 | <0.001 |
| Schoorl (2022) [41] | Netherlands | 39 | 27-30 weeks’ gestation | suspected IDE ** | Ret-He | Sysmex XN 9000 | ferrous fumarate 200 mg qd (100 mg Fe/day), 4 weeks | ·· | 25.6 ± 3.0 | 31.6 ± 2.6 | <0.001 |
| Akshaykirthan (2025) [51] | India | 315 | 12-16 weeks’ gestation | IDA (Hb = 7-9.9 g/dl, serum ferritin < 30 ng/mL and/or TSAT < 20%) | Ret-Hb | Sysmex | iron (60 mg of ferrous sulphate) and folic acid (400 mcg) tablets bid, to 26-30 weeks’ gestation | ·· | 23.30 ± 3.03 | 27.84 ± 3.83 | <0.001 |
