# Supplementary material for: Clinical applications of reticulocyte hemoglobin indices, including reticulocyte hemoglobin equivalent (Ret-He) and reticulocyte hemoglobin content (CHr), among pregnant and postpartum women: A scoping review and evidence map
Source: PLoS One. 2026 Nov 13;21(11):e0362481. doi: 10.1371/journal.pone.0362481 (PMC13766218; doi:10.1371/journal.pone.0362481)
Supplement: Supplementary file 14 [file pone.0362481.s013.docx]

S13 Tables. Changes in Ret-He/CHr value before and after iron-based interventions

A. Changes in MCHr before and after iron-based intervention in pregnant women with β-thalassemia trait (Breymann et al., 1999)

| Author (year) | Country | Study Design | N | T | Participants | Intervention | Parameter | Analyzers | Comparison group | Before (pg) | After (pg) | P value |
| --- | --- | --- | --- | --- | --- | --- | --- | --- | --- | --- | --- | --- |
| Breymann (1999) [19] | Switzerland | case series | 4 | 22-28 weeks’ gestation | β-thalassemia trait with anemia | RhEPO(300 U/kg b.w.) and iron III saccharate 100 mg i.v. | MCHr | Technicon H3 | patient1 (n = 1) | 20.4 | 20.9 | ·· |
| Breymann (1999) [19] | Switzerland | case series | 4 | 22-28 weeks’ gestation | β-thalassemia trait with anemia | RhEPO(300 U/kg b.w.) and iron III saccharate 100 mg i.v. | MCHr | Technicon H3 | patient2 (n = 1) | 20.1 | 20.8 | ·· |
| Breymann (1999) [19] | Switzerland | case series | 4 | 22-28 weeks’ gestation | β-thalassemia trait with anemia | RhEPO(300 U/kg b.w.) and iron III saccharate 100 mg i.v. | MCHr | Technicon H3 | patient3 (n = 1) | 19.6 | 20.2 | ·· |
| Breymann (1999) [19] | Switzerland | case series | 4 | 22-28 weeks’ gestation | β-thalassemia trait with anemia | RhEPO(300 U/kg b.w.) and iron III saccharate 100 mg i.v. | MCHr | Technicon H3 | patient4 (n = 1) | 20.2 | 19.9 | ·· |

B. Changes in MCHr before and after postpartum iron supplementation in women with antenatal non-anemic iron deficiency (Krafft et al., 2005)

| Author (year) | Country | Study design | N | T | Participants | Intervention | Parameter | Analyzers | Comparison group | Before (pg) | After (pg) | P value |
| --- | --- | --- | --- | --- | --- | --- | --- | --- | --- | --- | --- | --- |
| Krafft (2005) [22] | Switzerland | RCT | 52 | postpartum | antenatal nonanemic ID (ferritin <15, Hb >11); no postnatal anaemia (Hb >10) | iron sulphate 80 mg /day for 12 weeks | MCHr | Technicon H3 | iron sulphate (n = 28) | 27.0 ± 1.7 | 27.9 ± 1.1 | ·· |
| Krafft (2005) [22] | Switzerland | RCT | 52 | postpartum | antenatal nonanemic ID (ferritin <15, Hb >11); no postnatal anaemia (Hb >10) | placebo for 12 weeks | MCHr | Technicon H3 | placebo (n = 24) | 27.3 ± 1.9 | 27.6 ± 1.5 | ·· |

C. Changes in CHr before and after iron-based treatment in pregnant women with moderate anemia (Bencaiova et al., 2017)

| Author (year) | Country | Study design | N | T | Participants | Intervention | Parameter | Analyzers | Comparison group | Before (pg) | After (pg) | P value |
| --- | --- | --- | --- | --- | --- | --- | --- | --- | --- | --- | --- | --- |
| Amstad Bencaiova (2017) [31] | Switzerland | clinical trial | 50 | 19-38 weeks’ gestation | moderate anemia (Hb: 8.0-9.9, ferritin <15) | 200mg iron sucrose ±RhEPO (10,000 U) i.v. | CHr | Advia | IDA (baseline CHr <28 pg, n = 38) | 25.7 ± 1.2 | 32.5 ± 0.6 | 0.001 |
| Amstad Bencaiova (2017) [31] | Switzerland | clinical trial | 50 | 19-38 weeks’ gestation | moderate anemia (Hb: 8.0-9.9, ferritin <15) | 200mg iron sucrose ±RhEPO (10,000 U) i.v. | CHr | Advia | ACD＋ID (baseline CHr >28 pg, n = 12) | 30.6 ± 2.6 | 33.8 ± 1.0 | 0.014 |

D. Changes in RET-He before and after iron-based intervention with or without adjunctive supplementation in pregnant women with mild to moderate anemia (Afifah et al., 2025)

| Author (year) | Country | Study design | N | T | Participants | Intervention | Parameter | Analyzers | Comparison group | Before (pg) | After (pg) | P value |
| --- | --- | --- | --- | --- | --- | --- | --- | --- | --- | --- | --- | --- |
| Afifah (2025) [48] | Indonesia | quasi-experimental design | 40 | 14-32 weeks’ gestation | mild to moderate anemia (Hb: 7–10.9 ) | ferrous fumarate (60 mg Fe) and folic acid 400 μg bid for 2 weeks and fiber supplement (intervention) | RET-He | Sysmex series | intervention, baseline RET-He <32.1 pg (n = 14) | 29.2 (21-31.8) | 31.5 (20.3-34.5) | 0.087 |
| Afifah (2025) [48] | Indonesia | quasi-experimental design | 40 | 14-32 weeks’ gestation | mild to moderate anemia (Hb: 7–10.9 ) | ferrous fumarate (60 mg Fe) and folic acid 400 μg bid for 2 weeks and fiber supplement (intervention) | RET-He | Sysmex series | intervention, baseline RET-He ≥32.1 pg (n = 11) | 33.2 (32.1-35.1) | 32.1 (30.5-33.1) | 0.018 |
| Afifah (2025) [48] | Indonesia | quasi-experimental design | 40 | 14-32 weeks’ gestation | mild to moderate anemia (Hb: 7–10.9 ) | ferrous fumarate (60 mg Fe) and folic acid 400 μg bid for 2 weeks and milk powder (control) | RET-He | Sysmex series | control, baseline RET-He <32.1 pg (n = 8) | 27.75 (20.6-29.7) | 26.55 (20.3-31.9) | 0.674 |
| Afifah (2025) [48] | Indonesia | quasi-experimental design | 40 | 14-32 weeks’ gestation | mild to moderate anemia (Hb: 7–10.9 ) | ferrous fumarate (60 mg Fe) and folic acid 400 μg bid for 2 weeks and milk powder (control) | RET-He | Sysmex series | control, baseline RET-He ≥32.1 pg (n = 7) | 32.8 (32.1-35.2) | 31.6 (30.2-33.8) | 0.008 |

E. Changes in RET-He before and after iron-based intervention in pregnant women with iron-deficiency anemia (Patki et al., 2025)

| Author (year) | Country | Study design | N | T | Participants | Intervention | Parameter | Analyzers | Comparison group | Before (pg) | After (pg) | P value |
| --- | --- | --- | --- | --- | --- | --- | --- | --- | --- | --- | --- | --- |
| Patki (2025) [52] | India | randomized comparative study | 108 | 13-16 weeks’ gestation | iron-deficiency anemia (Hb 9–10.5 g/dL, and ferritin < 15 mcg/L) | SunActive™ Fe (27 mg of elemental iron) once daily for 4 weeks | RET-He | ·· | test, n = 55 | 25.98 ± 1.25 | 31.30 ± 3.10 | 0.001 |
| Patki (2025) [52] | India | randomized comparative study | 108 | 13-16 weeks’ gestation | iron-deficiency anemia (Hb 9–10.5 g/dL, and ferritin < 15 mcg/L) | Ferrous Ascorbate (100 mg of elemental iron) once daily for 4 weeks | RET-He | ·· | control, n = 56 | 26.22 ± 1.15 | 30.47 ± 3.09 | 0.001 |
