# Supplementary material for: Clinical applications of reticulocyte hemoglobin indices, including reticulocyte hemoglobin equivalent (Ret-He) and reticulocyte hemoglobin content (CHr), among pregnant and postpartum women: A scoping review and evidence map
Source: PLoS One. 2026 Nov 13;21(11):e0362481. doi: 10.1371/journal.pone.0362481 (PMC13766218; doi:10.1371/journal.pone.0362481)
Supplement: Supplementary file 15 [file pone.0362481.s016.docx]

We thank the editor, journal office, and reviewers for their careful evaluation of our manuscript and for their constructive comments. We have revised the manuscript accordingly. Our point-by-point responses are provided below.

**Response to Journal Requirements**

1. PLOS ONE style and file naming
   We have checked the manuscript, figure files, and Supporting Information files to ensure that they meet PLOS ONE style requirements, including file naming requirements.
2. Competing Interests Statement
   As requested, we have included the amended Competing Interests Statement in the resubmission cover letter. The statement includes confirmation that the declared competing interests do not alter our adherence to PLOS ONE policies on sharing data and materials.
3. References to Figures 1 and 3 in the text
   We have revised the Results section to ensure that Figures 1 and 3 are explicitly cited in the manuscript text.
4. Figure captions
   We have included a separate caption for each figure in the manuscript.
5. Previously published works recommended by reviewers
   No reviewer specifically recommended citation of any particular previously published work. We reviewed the relevant literature while addressing the reviewer comments and revised the manuscript using the existing references where appropriate. We determined that no additional references were necessary.

**Response to Reviewer Comments**

**Reviewer 1**
**Comments 1**. Line 80. Clarify the effect of inflammation (esp chronic inflammation) on RetHe and CHr as they relate to hepcidin levels in chronic inflammation.
**Comment 2.** Line 88. Clarify the relationship between iron supplementation and RetHe and CHr in pregnancy.

**Response to Comments 1 and 2:** We thank the reviewer for these helpful comments. We have revised the Introduction to clarify the relationship between inflammation and Ret-He/CHr. Specifically, we now state that Ret-He/CHr may decrease in inflammatory states when inflammatory cytokines and hepcidin restrict iron absorption and macrophage iron release, thereby limiting iron availability for erythropoiesis. We have also clarified that, unlike ferritin, Ret-He/CHr is not an acute-phase reactant and is not expected to increase directly in response to inflammation. In addition, we revised the objective statement to explicitly include the use of Ret-He/CHr for monitoring the response to iron therapy during pregnancy and the postpartum period.

**Reviewer 2**
**Comment 1.** Title, please consider changing the title more towards what the outcome of the review is

**Response:** We thank the reviewer for this suggestion. We carefully considered whether to revise the title to more directly reflect the review findings. However, because this is a scoping review and evidence map, the findings are primarily intended to describe the scope, distribution, and gaps in the existing evidence rather than to provide a definitive conclusion regarding diagnostic performance or clinical implementation. We therefore retained the current title, which reflects the population, indices of interest, and review design without overstating the certainty of the findings. To address the reviewer’s concern, we have revised the Discussion to more clearly describe the main implications of the review, including the current limitations of the evidence and the studies needed before implementation in routine perinatal care.

**Comment 2.** Regret that the findings are not conclusive and this methods remains hypothetical. Would the authors be able to be more specific and outline what would be needed in order to implement these new markers.

**Response:** We thank the reviewer for this important comment. We agree that the current evidence is not sufficient to support the universal implementation of Ret-He/CHr in routine perinatal care. We have revised the Discussion to more specifically outline what would be required before implementation. In particular, we now state that further studies are needed to establish population-, pregnancy-stage-, and analyzer-specific thresholds; evaluate whether Ret-He/CHr values or trajectories improve the prediction of clinically relevant maternal or neonatal outcomes; and determine how these indices should be integrated with existing markers such as hemoglobin and ferritin. We also emphasize the need for pragmatic longitudinal studies embedded in antenatal and postpartum care pathways.

**Comment 3.** Would the authors be able to comment about the ethnical difference of the Ret-He and CHr during pregnancy. How about undiagnosed sickle trait or thalassaemia carrier status ?

**Response:** We thank the reviewer for raising this important point. We have revised the Discussion to address ethnic or ancestry-related differences and the potential influence of undiagnosed hemoglobinopathy carrier status. We now state that ethnic or ancestry-related differences in Ret-He/CHr during pregnancy have not been well established. We also added that conditions impairing erythropoiesis or hemoglobin synthesis, including nutritional deficiencies such as vitamin B12 or folate deficiency and hemoglobinopathy carrier states such as thalassemia, may affect Ret-He/CHr values independently of iron deficiency. Accordingly, we now emphasize that the interpretation of Ret-He/CHr should take into account the characteristics of the target population, including nutritional status and the prevalence of hemoglobinopathy carrier states or other disorders affecting erythropoiesis. Because direct evidence on sickle cell trait and Ret-He/CHr during pregnancy is limited, we addressed this issue within the broader context of hemoglobinopathy carrier states.

**Comment 4.** There is a publication bias which should be addressed in the discussion.

**Response:** We thank the reviewer for this important comment. We have added publication and reporting biases as a limitation in the Discussion. Specifically, we now state that studies with favorable diagnostic performance, significant associations, or positive treatment responses may have been more likely to be published or reported in sufficient detail, and that the evidence map may therefore overrepresent applications with positive findings.

**Comment 5**. How feasible is it to get these values implemented in clinical practise ? Where does this fit in a care pathway; GP ? obstetrics ? etc.

**Response:** We thank the reviewer for this important comment. We have revised the Discussion to clarify the feasibility of implementing Ret-He/CHr and its potential position in care pathways. We now state that Ret-He/CHr may be feasible to implement in settings where hematology analyzers capable of measuring reticulocyte hemoglobin indices are already available, because these values can be obtained rapidly and at a relatively low cost as part of an extended complete blood count or reticulocyte analysis. We also describe potential care pathways, including early pregnancy screening, monitoring after the initiation of iron therapy, and postpartum reassessment. We note that Ret-He/CHr may be particularly useful in obstetric clinics, primary care settings, or resource-limited areas where immediate access to biochemical iron markers such as ferritin is difficult. We have further clarified that Ret-He/CHr may also serve as adjunctive markers in tertiary or hospital-based obstetric care when ferritin is difficult to interpret because of hemodilution, inflammation, or other clinical conditions.
