# Supplementary material for: Clinical applications of reticulocyte hemoglobin indices, including reticulocyte hemoglobin equivalent (Ret-He) and reticulocyte hemoglobin content (CHr), among pregnant and postpartum women: A scoping review and evidence map
Source: PLoS One. 2026 Nov 13;21(11):e0362481. doi: 10.1371/journal.pone.0362481 (PMC13766218; doi:10.1371/journal.pone.0362481)
Supplement: Supplementary file 16 [file pone.0362481.s017.docx]

We thank the Academic Editor and Reviewers for their careful evaluation of our revised manuscript. We appreciate their constructive comments and have addressed the remaining minor revision request as detailed below.

**Response to Journal Requirements**

**1. Citation of previously published works**

*Comment:* If the reviewer comments include a recommendation to cite specific previously published works, please review and evaluate these publications to determine whether they are relevant and should be cited.

**Response:** No Reviewer recommended citation of any specific previously published work in this round of review. No additional references were required to address the remaining comment.

**2. Reference list**

*Comment:* Please review your reference list to ensure that it is complete and correct, including the status of any retracted publications.

**Response:** We have reviewed the reference list for completeness and accuracy, including the retraction status of cited publications. No retracted references were identified. We have updated the bibliographic information for Patki et al. [52] and Pekar et al. [53]. In addition, we have added a footnote to Table 1 to clarify that the online publication year (2025) was used for Pekar et al. [53], as the article was available before the updated literature search.

**Response to the Academic Editor**

*Comment:* Reviewer 1 recommends one very minor edit to the text, one change in wording for a sentence.

**Response:** We thank the Academic Editor for their thoughtful recommendation. We have implemented the requested wording change in the Introduction, as detailed in our response to Reviewer 1 below.

**Response to Reviewer 1**

*Comment:* Line 66 - Ret-He and CHr are measured values, they are calculated from measured values. "which measure reticulocyte hemoglobin content" They assess rather than measure.

**Response:** We thank the Reviewer for their insightful clarification. We agree that Ret-He and CHr are calculated indices derived from instrument measurements rather than direct measurements of reticulocyte hemoglobin content. Accordingly, we have replaced “which measure reticulocyte hemoglobin content” with “which assess the hemoglobin content of reticulocytes” in the Introduction.

**Response to Reviewer 2**

*Comment:* All queries were fully addressed.

**Response:** We thank the Reviewer for their careful evaluation of our revised manuscript and for confirming that all previous concerns have been addressed.
