# Supplementary material for: Clinical applications of reticulocyte hemoglobin indices, including reticulocyte hemoglobin equivalent (Ret-He) and reticulocyte hemoglobin content (CHr), among pregnant and postpartum women: A scoping review and evidence map
Source: PLoS One. 2026 Nov 13;21(11):e0362481. doi: 10.1371/journal.pone.0362481 (PMC13766218; doi:10.1371/journal.pone.0362481)
Supplement: Peer review history

# Peer review: Decision Letter 0

**DOI:** 10.1371/journal.pone.0362481.r001

**Authors:** Regina Day

20 Aug 2026

Dear Dr. Ohsuga,

Thank you for submitting your manuscript to PLOS One. After careful consideration, we feel that it has merit but does not fully meet PLOS One’s publication criteria as it currently stands. Therefore, we invite you to submit a revised version of the manuscript that addresses the points raised during the review process.

Please submit your revised manuscript by Oct 19 2026 11:59PM. If you will need more time than this to complete your revisions, please reply to this message or contact the journal office at plosone@plos.org. When you're ready to submit your revision, log on to https://www.editorialmanager.com/pone/ and select the 'Submissions Needing Revision' folder to locate your manuscript file.

- A letter that responds to each point raised by the academic editor and reviewer(s). You should upload this letter as a separate file labeled 'Response to Reviewers'.
- A marked-up copy of your manuscript that highlights changes made to the original version. You should upload this as a separate file labeled 'Revised Manuscript with Track Changes'.
- An unmarked version of your revised paper without tracked changes. You should upload this as a separate file labeled 'Manuscript'.

If you would like to make changes to your financial disclosure, please include your updated statement in your cover letter. Guidelines for resubmitting your figure files are available below the reviewer comments at the end of this letter.

If applicable, we recommend that you deposit your laboratory protocols in protocols.io to enhance the reproducibility of your results. Protocols.io assigns your protocol its own identifier (DOI) so that it can be cited independently in the future. For instructions see: https://journals.plos.org/plosone/s/submission-guidelines#loc-laboratory-protocols. Additionally, PLOS One offers an option for publishing peer-reviewed Lab Protocol articles, which describe protocols hosted on protocols.io. Read more information on sharing protocols at https://plos.org/protocols?utm_medium=editorial-email&utm_source=authorletters&utm_campaign=protocols.

As the corresponding author, your ORCID iD is verified in the submission system and will appear in the published article. PLOS supports the use of ORCID, and we encourage all coauthors to register for an ORCID iD and use it as well. Please encourage your coauthors to verify their ORCID iD within the submission system before final acceptance, as unverified ORCID iDs will not appear in the published article. *Only*  the individual author can complete the verification step; PLOS staff *cannot*  verify ORCID iDs on behalf of authors.

We look forward to receiving your revised manuscript.

Kind regards,

Regina M. Day, Ph.D.

Academic Editor

PLOS One

**Journal Requirements:**

When submitting your revision, we need you to address these additional requirements.

1. Please ensure that your manuscript meets PLOS ONE's style requirements, including those for file naming. The PLOS ONE style templates can be found at

https://journals.plos.org/plosone/s/file?id=wjVg/PLOSOne_formatting_sample_main_body.pdf and

https://journals.plos.org/plosone/s/file?id=ba62/PLOSOne_formatting_sample_title_authors_affiliations.pdf

2. Thank you for stating the following in the Competing Interests section:

“I have read the journal's policy and the authors of this manuscript have the following competing interests: TO and ME have received research funding from Sysmex Corporation, a company involved in the development of reticulocyte hemoglobin–based indices, outside the submitted work; research funding from YStory Inc., outside the submitted work; and academic advisory fees from Tokio Marine Healthcare Co., Ltd. TO has received academic advisory fees from ITO EN, Ltd. YT is employed through a joint research fund between Kyoto University and HealthTech Laboratory Inc. TN reports grants or contracts from I&H Co., Ltd., Cocokarafine Group Co., Ltd., Konica Minolta, Inc., NTT DATA, Takeda Pharmaceutical Co., and Toppan Holdings Inc., outside the submitted work. TN reports consulting fees from Otsuka Pharmaceutical Co., Takeda Pharmaceutical Co., Johnson & Johnson K.K., and AstraZeneca plc; payment or honoraria for lectures, manuscript writing, or educational events from Pfizer Japan Inc., MSD K.K., Chugai Pharmaceutical Co., Takeda Pharmaceutical Co., Janssen Pharmaceutical K.K., Boehringer Ingelheim International GmbH, Eli Lilly Japan K.K., Maruho Co., Ltd., Mitsubishi Tanabe Pharma Co., Novartis Pharma K.K., Allergan Japan K.K., Novo Nordisk Pharma Ltd., TOA EIYO Ltd., AbbVie Inc., Ono Pharmaceutical Co., Ltd., GSK plc, Alexion Pharmaceuticals, Inc., Canon Medical Systems Co., Kowa Company, Limited, Araya, Merck & Co., Amicus Therapeutics, Inc., CSL Behring, Amgen Inc., Sanofi S.A., and BeOne Medicines I GmbH; stock options in BonBon Inc.; and donation support from Wiznet Co. The other authors have no competing interests to declare.”

We note that you received funding from a commercial source:

Sysmex Corporation, YStory Inc., Tokio Marine Healthcare Co., Ltd., HealthTech Laboratory Inc., I&H Co., Ltd., Cocokarafine Group Co., Ltd., Konica Minolta, Inc., Takeda Pharmaceutical Co., and Toppan Holdings Inc., Otsuka Pharmaceutical Co., Johnson & Johnson K.K., and AstraZeneca plc, Pfizer Japan Inc, Chugai Pharmaceutical Co., Janssen Pharmaceutical, Boehringer Ingelheim International GmbH, Eli Lilly Japan K.K., Maruho Co., Ltd., Mitsubishi Tanabe Pharma Co., Novartis Pharma K.K., Allergan Japan K.K., Novo Nordisk Pharma Ltd., TOA EIYO Ltd., AbbVie Inc., Ono Pharmaceutical Co., Ltd., GSK plc, Alexion Pharmaceuticals, Inc., Canon Medical Systems Co., Kowa Company, Limited, Araya, Merck & Co., Amicus Therapeutics, Inc., CSL Behring, Amgen Inc., Sanofi S.A., and BeOne Medicines I GmbH, BonBon Inc, Wiznet Co,

Please provide an amended Competing Interests Statement that explicitly states this commercial funder, along with any other relevant declarations relating to employment, consultancy, patents, products in development, marketed products, etc.

Within this Competing Interests Statement, please confirm that this does not alter your adherence to all PLOS ONE policies on sharing data and materials by including the following statement: "This does not alter our adherence to PLOS ONE policies on sharing data and materials.” (as detailed online in our guide for authors http://journals.plos.org/plosone/s/competing-interests).  If there are restrictions on sharing of data and/or materials, please state these. Please note that we cannot proceed with consideration of your article until this information has been declared.

Please include your amended Competing Interests Statement within your cover letter. We will change the online submission form on your behalf..

3. Please ensure that you refer to Figures 1 and 3 in your text as, if accepted, production will need this reference to link the reader to the figure.

4. Please include a separate caption for each figure in your manuscript.

5. If the reviewer comments include a recommendation to cite specific previously published works, please review and evaluate these publications to determine whether they are relevant and should be cited. There is no requirement to cite these works unless the editor has indicated otherwise.

**Additional Editor Comments:**

Two Reviewers have evaluated the manuscript "Clinical applications of reticulocyte hemoglobin indices, including reticulocyte hemoglobin equivalent (Ret-He) and reticulocyte hemoglobin content (CHr), among pregnant and postpartum women: a scoping review and evidence map". Although the Reviewers find the manuscript overall is well written, several questions remain for the authors to address. Please consider the comments of the Reviewers and address each concern.

Reviewer 1 comments:

The review is well written and has an important message, but no clear advise or future plan has been provided. There is no change in management provided. Therefore we are stuck with out old methods of measuring IDA in pregnant woman. Overall, this paper brings up a novel use for RetHe/CHr in pregnancy, but the disparate studies reviewed made the data somewhat challenging to interpret, which the authors acknowledge in their manuscript.

Recommended these edits to the author:

Line 80. Clarify the effect of inflammation (esp chronic inflammation) on RetHe and CHr as they relate to hepcidin levels in chronic inflammation.

Line 88. Clarify the relationship between iron supplementation and RetHe and CHr in pregnancy.

Reviewer 2 comments:

The authors present a well written review and evidence map around picking up IDA and ID in pregnant woman using Ret-He and CHr. I have several comments and questions.

1. Title, please consider changing the title more towards what the outcome of the review is

2. Regret that the findings are not conclusive and this methods remains hypothetical. Would the authors be able to be more specific and outline what would be needed in order to implement these new markers.

3. Would the authors be able to comment about the ethnical difference of the Ret-He and CHr during pregnancy. How about undiagnosed sickle trait or thalassaemia carrier status ?

4. There is a publication bias which should be addressed in the discussion.

5. How feasible is it to get these values implemented in clinical practise ? Where does this fit in a care pathway; GP ? obstetrics ? etc.

Best of luck with this manuscript.

[Note: HTML markup is below. Please do not edit.]

Reviewers' comments:

Reviewer's Responses to Questions

**Comments to the Author**

1. Is the manuscript technically sound, and do the data support the conclusions?

Reviewer #1: Yes

Reviewer #2: No

**********

2. Has the statistical analysis been performed appropriately and rigorously?

Reviewer #1: Yes

Reviewer #2: Yes

**********

3. Have the authors made all data underlying the findings in their manuscript fully available?

The PLOS Data policy

Reviewer #1: Yes

Reviewer #2: Yes

**********

4. Is the manuscript presented in an intelligible fashion and written in standard English?

Reviewer #1: Yes

Reviewer #2: Yes

**********

Reviewer #1: Line 80. Clarify the effect of inflammation (esp chronic inflammation) on RetHe and CHr as they relate to hepcidin levels in chronic inflammation.

Line 88. Clarify the relationship between iron supplementation and RetHe and CHr in pregnancy.

Reviewer #2: The authors present a well written review and evidence map around picking up IDA and ID in pregnant woman using Ret-He and CHr. I have several comments and questions.

1. Title, please consider changing the title more towards what the outcome of the review is

2. Regret that the findings are not conclusive and this methods remains hypothetical. Would the authors be able to be more specific and outline what would be needed in order to implement these new markers.

3. Would the authors be able to comment about the ethnical difference of the Ret-He and CHr during pregnancy. How about undiagnosed sickle trait or thalassaemia carrier status ?

4. There is a publication bias which should be addressed in the discussion.

5. How feasible is it to get these values implemented in clinical practise ? Where does this fit in a care pathway; GP ? obstetrics ? etc.

Best of luck with this manuscript.

**********

what does this mean?). If published, this will include your full peer review and any attached files.

If you choose “no”, your identity will remain anonymous but your review may still be made public.

**Do you want your identity to be public for this peer review?**  For information about this choice, including consent withdrawal, please see our Privacy Policy

Reviewer #1: No

Reviewer #2: **Yes:**  Dr Sanne Lugthart

**********

[NOTE: If reviewer comments were submitted as an attachment file, they will be attached to this email and accessible via the submission site. Please log into your account, locate the manuscript record, and check for the action link "View Attachments". If this link does not appear, there are no attachment files.]

To ensure your figures meet our technical requirements, please review our figure guidelines: https://journals.plos.org/plosone/s/figures

You may also use PLOS’s free figure tool, NAAS, to help you prepare publication quality figures: https://journals.plos.org/plosone/s/figures#loc-tools-for-figure-preparation.

NAAS will assess whether your figures meet our technical requirements by comparing each figure against our figure specifications.

# Author comment: Author response to Decision Letter 1

**DOI:** 10.1371/journal.pone.0362481.r002

4 Oct 2026

Reviewer 1

Comments 1. Line 80. Clarify the effect of inflammation (esp chronic inflammation) on RetHe and CHr as they relate to hepcidin levels in chronic inflammation.

Comment 2. Line 88. Clarify the relationship between iron supplementation and RetHe and CHr in pregnancy.

Response to Comments 1 and 2: We thank the reviewer for these helpful comments. We have revised the Introduction to clarify the relationship between inflammation and Ret-He/CHr. Specifically, we now state that Ret-He/CHr may decrease in inflammatory states when inflammatory cytokines and hepcidin restrict iron absorption and macrophage iron release, thereby limiting iron availability for erythropoiesis. We have also clarified that, unlike ferritin, Ret-He/CHr is not an acute-phase reactant and is not expected to increase directly in response to inflammation. In addition, we revised the objective statement to explicitly include the use of Ret-He/CHr for monitoring the response to iron therapy during pregnancy and the postpartum period.

Reviewer 2

Comment 1. Title, please consider changing the title more towards what the outcome of the review is

Response: We thank the reviewer for this suggestion. We carefully considered whether to revise the title to more directly reflect the review findings. However, because this is a scoping review and evidence map, the findings are primarily intended to describe the scope, distribution, and gaps in the existing evidence rather than to provide a definitive conclusion regarding diagnostic performance or clinical implementation. We therefore retained the current title, which reflects the population, indices of interest, and review design without overstating the certainty of the findings. To address the reviewer’s concern, we have revised the Discussion to more clearly describe the main implications of the review, including the current limitations of the evidence and the studies needed before implementation in routine perinatal care.

Comment 2. Regret that the findings are not conclusive and this methods remains hypothetical. Would the authors be able to be more specific and outline what would be needed in order to implement these new markers.

Response: We thank the reviewer for this important comment. We agree that the current evidence is not sufficient to support the universal implementation of Ret-He/CHr in routine perinatal care. We have revised the Discussion to more specifically outline what would be required before implementation. In particular, we now state that further studies are needed to establish population-, pregnancy-stage-, and analyzer-specific thresholds; evaluate whether Ret-He/CHr values or trajectories improve the prediction of clinically relevant maternal or neonatal outcomes; and determine how these indices should be integrated with existing markers such as hemoglobin and ferritin. We also emphasize the need for pragmatic longitudinal studies embedded in antenatal and postpartum care pathways.

Comment 3. Would the authors be able to comment about the ethnical difference of the Ret-He and CHr during pregnancy. How about undiagnosed sickle trait or thalassaemia carrier status ?

Response: We thank the reviewer for raising this important point. We have revised the Discussion to address ethnic or ancestry-related differences and the potential influence of undiagnosed hemoglobinopathy carrier status. We now state that ethnic or ancestry-related differences in Ret-He/CHr during pregnancy have not been well established. We also added that conditions impairing erythropoiesis or hemoglobin synthesis, including nutritional deficiencies such as vitamin B12 or folate deficiency and hemoglobinopathy carrier states such as thalassemia, may affect Ret-He/CHr values independently of iron deficiency. Accordingly, we now emphasize that the interpretation of Ret-He/CHr should take into account the characteristics of the target population, including nutritional status and the prevalence of hemoglobinopathy carrier states or other disorders affecting erythropoiesis. Because direct evidence on sickle cell trait and Ret-He/CHr during pregnancy is limited, we addressed this issue within the broader context of hemoglobinopathy carrier states.

Comment 4. There is a publication bias which should be addressed in the discussion.

Response: We thank the reviewer for this important comment. We have added publication and reporting biases as a limitation in the Discussion. Specifically, we now state that studies with favorable diagnostic performance, significant associations, or positive treatment responses may have been more likely to be published or reported in sufficient detail, and that the evidence map may therefore overrepresent applications with positive findings.

Comment 5. How feasible is it to get these values implemented in clinical practise ? Where does this fit in a care pathway; GP ? obstetrics ? etc.

Response: We thank the reviewer for this important comment. We have revised the Discussion to clarify the feasibility of implementing Ret-He/CHr and its potential position in care pathways. We now state that Ret-He/CHr may be feasible to implement in settings where hematology analyzers capable of measuring reticulocyte hemoglobin indices are already available, because these values can be obtained rapidly and at a relatively low cost as part of an extended complete blood count or reticulocyte analysis. We also describe potential care pathways, including early pregnancy screening, monitoring after the initiation of iron therapy, and postpartum reassessment. We note that Ret-He/CHr may be particularly useful in obstetric clinics, primary care settings, or resource-limited areas where immediate access to biochemical iron markers such as ferritin is difficult. We have further clarified that Ret-He/CHr may also serve as adjunctive markers in tertiary or hospital-based obstetric care when ferritin is difficult to interpret because of hemodilution, inflammation, or other clinical conditions.

Attachment Submitted filename: Response to Reviewers1005.docx

# Peer review: Decision Letter 1

**DOI:** 10.1371/journal.pone.0362481.r003

**Authors:** Regina Day

14 Oct 2026

Dear Dr. Ohsuga,

Thank you for submitting your manuscript to PLOS One. After careful evaluation of the revised manuscript, there remains one recommendation for minor revision of the manuscript. Therefore, we invite you to submit a revised version of the manuscript that addresses the points raised during the review process.

==============================

Reviewer 1 recommends a minor change in the text of the manuscript:

Line 66 - Ret-He and CHr are measured values, they are calculated from measured values. "which measure reticulocyte hemoglobin content" They assess rather than measure.

Please submit your revised manuscript by Nov 13 2026 11:59PM. If you will need more time than this to complete your revisions, please reply to this message or contact the journal office at plosone@plos.org. When you're ready to submit your revision, log on to https://www.editorialmanager.com/pone/ and select the 'Submissions Needing Revision' folder to locate your manuscript file.

- A letter that responds to each point raised by the academic editor and reviewer(s). You should upload this letter as a separate file labeled 'Response to Reviewers'.
- A marked-up copy of your manuscript that highlights changes made to the original version. You should upload this as a separate file labeled 'Revised Manuscript with Track Changes'.
- An unmarked version of your revised paper without tracked changes. You should upload this as a separate file labeled 'Manuscript'.

If you would like to make changes to your financial disclosure, please include your updated statement in your cover letter. Guidelines for resubmitting your figure files are available below the reviewer comments at the end of this letter.

If applicable, we recommend that you deposit your laboratory protocols in protocols.io to enhance the reproducibility of your results. Protocols.io assigns your protocol its own identifier (DOI) so that it can be cited independently in the future. For instructions see: https://journals.plos.org/plosone/s/submission-guidelines#loc-laboratory-protocols. Additionally, PLOS One offers an option for publishing peer-reviewed Lab Protocol articles, which describe protocols hosted on protocols.io. Read more information on sharing protocols at https://plos.org/protocols?utm_medium=editorial-email&utm_source=authorletters&utm_campaign=protocols.

As the corresponding author, your ORCID iD is verified in the submission system and will appear in the published article. PLOS supports the use of ORCID, and we encourage all coauthors to register for an ORCID iD and use it as well. Please encourage your coauthors to verify their ORCID iD within the submission system before final acceptance, as unverified ORCID iDs will not appear in the published article. *Only*  the individual author can complete the verification step; PLOS staff *cannot*  verify ORCID iDs on behalf of authors.

We look forward to receiving your revised manuscript.

Kind regards,

Regina M. Day, Ph.D.

Academic Editor

PLOS One

Journal Requirements:

1. If the reviewer comments include a recommendation to cite specific previously published works, please review and evaluate these publications to determine whether they are relevant and should be cited. There is no requirement to cite these works unless the editor has indicated otherwise.

2. Please review your reference list to ensure that it is complete and correct. If you have cited papers that have been retracted, please include the rationale for doing so in the manuscript text, or remove these references and replace them with relevant current references. Any changes to the reference list should be mentioned in the rebuttal letter that accompanies your revised manuscript. If you need to cite a retracted article, indicate the article’s retracted status in the References list and also include a citation and full reference for the retraction notice.

Additional Editor Comments:

Reviewer 1 recommends one very minor edit to the text, one change in wording for a sentence.

[Note: HTML markup is below. Please do not edit.]

Reviewers' comments:

Reviewer's Responses to Questions

**Comments to the Author**

Reviewer #1: All comments have been addressed

Reviewer #2: All comments have been addressed

**********

2. Is the manuscript technically sound, and do the data support the conclusions?

Reviewer #1: Yes

Reviewer #2: Partly

**********

3. Has the statistical analysis been performed appropriately and rigorously?

Reviewer #1: I Don't Know

Reviewer #2: Yes

**********

4. Have the authors made all data underlying the findings in their manuscript fully available?

The PLOS Data policy

Reviewer #1: Yes

Reviewer #2: Yes

**********

5. Is the manuscript presented in an intelligible fashion and written in standard English?

Reviewer #1: Yes

Reviewer #2: Yes

**********

Reviewer #1: Line 66 - Ret-He and CHr are measured values, they are calculated from measured values. "which measure reticulocyte hemoglobin content" They assess rather than measure.

Reviewer #2: Thank you for resubmitting a revised version of your manuscript.

All queries were fully addressed.

I wish you the best of luck with this submission.

**********

what does this mean?). If published, this will include your full peer review and any attached files.

If you choose “no”, your identity will remain anonymous but your review may still be made public.

**Do you want your identity to be public for this peer review?**  For information about this choice, including consent withdrawal, please see our Privacy Policy

Reviewer #1: No

Reviewer #2: **Yes:**  Dr Sanne Lugthart

**********

[NOTE: If reviewer comments were submitted as an attachment file, they will be attached to this email and accessible via the submission site. Please log into your account, locate the manuscript record, and check for the action link "View Attachments". If this link does not appear, there are no attachment files.]

To ensure your figures meet our technical requirements, please review our figure guidelines: https://journals.plos.org/plosone/s/figures

You may also use PLOS’s free figure tool, NAAS, to help you prepare publication quality figures: https://journals.plos.org/plosone/s/figures#loc-tools-for-figure-preparation.

NAAS will assess whether your figures meet our technical requirements by comparing each figure against our figure specifications.

# Author comment: Author response to Decision Letter 2

**DOI:** 10.1371/journal.pone.0362481.r004

23 Oct 2026

Response to the Academic Editor

Comment: Reviewer 1 recommends one very minor edit to the text, one change in wording for a sentence.

Response: We thank the Academic Editor for their thoughtful recommendation. We have implemented the requested wording change in the Introduction, as detailed in our response to Reviewer 1 below.

Response to Reviewer 1

Comment: Line 66 - Ret-He and CHr are measured values, they are calculated from measured values. "which measure reticulocyte hemoglobin content" They assess rather than measure.

Response: We thank the Reviewer for their insightful clarification. We agree that Ret-He and CHr are calculated indices derived from instrument measurements rather than direct measurements of reticulocyte hemoglobin content. Accordingly, we have replaced “which measure reticulocyte hemoglobin content” with “which assess the hemoglobin content of reticulocytes” in the Introduction.

Response to Reviewer 2

Comment: All queries were fully addressed.

Response: We thank the Reviewer for their careful evaluation of our revised manuscript and for confirming that all previous concerns have been addressed.

Attachment Submitted filename: Response to Reviewers2.docx

# Editor report: Decision Letter 2

**DOI:** 10.1371/journal.pone.0362481.r005

**Authors:** Regina Day

26 Oct 2026

Clinical applications of reticulocyte hemoglobin indices, including reticulocyte hemoglobin equivalent (Ret-He) and reticulocyte hemoglobin content (CHr), among pregnant and postpartum women: a scoping review and evidence map

PONE-D-26-35769R2

Dear Dr. Ohsuga,

We’re pleased to inform you that your manuscript has been judged scientifically suitable for publication and will be formally accepted for publication once it meets all outstanding technical requirements.

Within one week, you’ll receive an e-mail detailing the required amendments. When these have been addressed, you’ll receive a formal acceptance letter and your manuscript will be scheduled for publication.

An invoice will be generated when your article is formally accepted. Please note, if your institution has a publishing partnership with PLOS and your article meets the relevant criteria, all or part of your publication costs will be covered. Please make sure your user information is up-to-date by logging into Editorial Manager at Editorial Manager® and clicking the ‘Update My Information' link at the top of the page. For questions related to billing, please contact billing support.

If your institution or institutions have a press office, please notify them about your upcoming paper to help maximize its impact. If they’ll be preparing press materials, please inform our press team as soon as possible -- no later than 48 hours after receiving the formal acceptance. Your manuscript will remain under strict press embargo until 2 pm Eastern Time on the date of publication. For more information, please contact onepress@plos.org.

Kind regards,

Regina M. Day, Ph.D.

Academic Editor

PLOS One

Additional Editor Comments (optional):

All comments have been addressed

Reviewers' comments:

# Editor report: Acceptance letter

**DOI:** 10.1371/journal.pone.0362481.r006

**Authors:** Regina Day

PONE-D-26-35769R2

PLOS One

Dear Dr. Ohsuga,

I'm pleased to inform you that your manuscript has been deemed suitable for publication in PLOS One. Congratulations! Your manuscript is now being handed over to our production team.

At this stage, our production department will prepare your paper for publication. This includes ensuring the following:

* All references, tables, and figures are properly cited

* All relevant supporting information is included in the manuscript submission,

* There are no issues that prevent the paper from being properly typeset

You will receive further instructions from the production team, including instructions on how to review your proof when it is ready. Please keep in mind that we are working through a large volume of accepted articles, so please give us a few days to review your paper and let you know the next and final steps.

Lastly, if your institution or institutions have a press office, please let them know about your upcoming paper now to help maximize its impact. If they'll be preparing press materials, please inform our press team within the next 48 hours. Your manuscript will remain under strict press embargo until 2 pm Eastern Time on the date of publication. For more information, please contact onepress@plos.org.

You will receive an invoice from PLOS for your publication fee after your manuscript has reached the completed accept phase. If you receive an email requesting payment before acceptance or for any other service, this may be a phishing scheme. Learn how to identify phishing emails and protect your accounts at https://explore.plos.org/phishing.

If we can help with anything else, please email us at customercare@plos.org.

Thank you for submitting your work to PLOS One and supporting open access.

Kind regards,

PLOS One Editorial Office Staff

on behalf of

Dr. Regina M. Day

Academic Editor

PLOS One
